# The silencing of *ets-4* mRNA relies on the functional cooperation between REGE-1/Regnase-1 and RLE-1/Roquin-1

**DOI:** 10.1093/nar/gkac609

**Published:** 2022-07-12

**Authors:** Daria Sobańska, Alicja A Komur, Agnieszka Chabowska-Kita, Julita Gumna, Pooja Kumari, Katarzyna Pachulska-Wieczorek, Rafal Ciosk

**Affiliations:** Institute of Bioorganic Chemistry, Polish Academy of Sciences, Poznań 61-704, Poland; Institute of Bioorganic Chemistry, Polish Academy of Sciences, Poznań 61-704, Poland; Institute of Bioorganic Chemistry, Polish Academy of Sciences, Poznań 61-704, Poland; Institute of Bioorganic Chemistry, Polish Academy of Sciences, Poznań 61-704, Poland; Department of Biosciences, University of Oslo, Oslo 0316, Norway; Institute of Bioorganic Chemistry, Polish Academy of Sciences, Poznań 61-704, Poland; Institute of Bioorganic Chemistry, Polish Academy of Sciences, Poznań 61-704, Poland; Department of Biosciences, University of Oslo, Oslo 0316, Norway

## Abstract

Regnase-1 is an evolutionarily conserved endoribonuclease. It degrades diverse mRNAs important for many biological processes including immune homeostasis, development and cancer. There are two competing models of Regnase-1-mediated mRNA silencing. One model postulates that Regnase-1 works together with another RNA-binding protein, Roquin-1, which recruits Regnase-1 to specific mRNAs. The other model proposes that the two proteins function separately. Studying REGE-1, the *Caenorhabditis elegans* ortholog of Regnase-1, we have uncovered its functional relationship with RLE-1, the nematode counterpart of Roquin-1. While both proteins are essential for mRNA silencing, REGE-1 and RLE-1 appear to associate with target mRNA independently of each other. Thus, although the functional interdependence between REGE-1/Regnase-1 and RLE-1/Roquin-1 is conserved, the underlying mechanisms may display species-specific variation, providing a rare perspective on the evolution of this important post-transcriptional regulatory mechanism.

## INTRODUCTION

mRNA is regulated at different levels, including at the level of transcript stability. For many mRNAs, the specificity of mRNA regulation is dictated by *cis*-acting RNA motifs that associate with diverse RNA-binding proteins (RBPs) and/or non-coding RNAs. An important class of RBPs affecting mRNA stability are RNases. RNases degrade RNA either from its ends (exoribonucleases) or via internal cleavages (endoribonucleases). A well-known example of the latter is the mammalian Regnase-1 (regulatory RNase 1), also known as Mcpip1 or Zc3h12a (1,2). Regnase-1 plays many biological roles, functioning in innate immunity, cancer, infection, angiogenesis, cell differentiation and apoptosis (3). Regnase-1 was initially reported to cooperate with another RBP, Roquin-1, which was suggested to recruit Regnase-1 to specific mRNA targets during T-cell activation (4). Consistent with this model, a recent study reported a physical interaction between these two proteins that is important for mRNA silencing (5). However, investigations in other cell types revealed that Regnase-1 can function independently of Roquin-1 (6,7). In the latter scenario, Regnase-1 degrades translationally active mRNAs with the help of Upf1, a helicase known for its role in nonsense-mediated RNA decay, which facilitates endonucleolytic cleavage by unwinding structured RNA (8).

Both Regnase-1 and Roquin-1 bind the 3′-untranslated regions (3′-UTRs) of target mRNAs (6). In RNA cleavage assays, Regnase-1 did not display a strong preference for RNA sequences or structures (9). Other studies suggested that Regnase-1 targets transcripts harboring stem–loops (SLs), and interfering with formation of these SLs abolishes the degradation (6). Whether these SLs recruit Regnase-1 remains unclear. Finally, according to the cooperative model, Regnase-1 is recruited to specific mRNAs by Roquin-1 (4,5). In contrast, the RNA binding of Roquin-1 and related proteins (collectively referred to as ‘Roquin’) is well understood. Roquin binds specific SLs, with the loops containing conserved motifs of either three nucleotides (called constitutive decay elements, CDEs) (10,11) or six nucleotides (alternative decay elements, ADEs) (11–13). The binding to these SLs is mediated by the ROQ domain of Roquin (13,14).

Recently, we characterized the *Caenorhabditis elegans* ortholog of Regnase-1, which we called REGE-1 (REGnasE-1) (15). REGE-1 and Regnase-1 display similar domain architecture and share residues critical for their RNase activity (16). Studying biological functions of REGE-1, we identified *ets-4* mRNA as its key target. ETS-4 is a conserved transcription factor involved in various aspects of nematode physiology, including the regulation of life span, body fat, cold resistance and possibly pathogen defense (15,17). Interestingly, while REGE-1 degrades *ets-4* mRNA, ETS-4 protein up-regulates (directly or indirectly) the transcription of *rege-1* (15). Thus, REGE-1 and ETS-4 form an autoregulatory axis influencing various aspects of animal biology. We previously demonstrated that a 115 nucleotide long fragment within the *ets-4* mRNA 3′-UTR is sufficient for the degradation by REGE-1 (15). However, the specificity of REGE-1 towards this fragment and the potential involvement of other players remained unknown. Dissecting the degradation of *ets-4* mRNA by REGE-1, we demonstrate here that it also requires the *C. elegans* counterpart of Roquin-1, RLE-1. While this is reminiscent of the cooperation between Regnase-1 and Roquin-1 observed in some studies (4,5), our findings suggest an independent recruitment of each protein to mRNA. Intriguingly, although Regnase-1 is known to target many diverse transcripts, our analysis suggests that *ets-4* mRNA may be the sole target of REGE-1. It suggests a model where REGE-1 controls various aspects of animal physiology by regulating the abundance of a single master regulator, ETS-4, which in turn controls the transcription of diverse effectors.

## MATERIALS AND METHODS

### Biological resources

All *C. elegans* strains used in these studies are listed in Supplementary Table S1.

Human embryonic kidney (HEK) 293T cells were obtained from the ATCC, USA (https://www.atcc.org/products/crl-3216).

### *C. elegans* handling and genetic manipulation

Animals were grown at 20°C on standard NGM plates, fed with the OP50 *Escherichia coli* (18). The CRISPR/Cas9 genome editing was performed by SunyBiotech (China) to: generate the *rle-1* ROQ domain mutant [allele *rrr61* (*syb517*)], C-terminally tag *rle-1* with green fluorescent protein (GFP)–FLAG [allele *rrr58* (*syb1279*)], C-terminally tag the *rle-1* ROQ domain mutant with GFP–FLAG [allele *rrr59* (*syb5530*)] and generate the C-terminally GFP–FLAG-tagged *rle-1 sanroque* mutant [allele *rrr62* (*syb5565*)]. The strains expressing the F1SΔADE reporter (allele *rrrSi500*) and the F1SΔRCE reporter (allele *sybSi111*) were generated in our lab and by SunyBiotech, respectively, by the MosSCI method utilizing the insertion locus ttTi5605.

RNA interference (RNAi) of individual genes was performed by feeding animals with bacteria expressing double-stranded RNA, beginning from the L1 larval stage until the young adult stage at 20°C, except for the RNAi of the CCR4–NOT deadenylase complex that was done from the L4 stage. The L4440 (empty) vector was used as a negative RNAi control. In this study, we used previously published RNAi clones: *ccr-4* (ZC518.3) (19), *ntl-1* (F57B9.2) (20), *rege-1* (C30F12.1) (15,21), *rle-1* (M142.6) (21), *dcap-1* (Y55F3A_748.c) (22), *dcap-2* (F52G2.1a) (22), *panl-2* (F31E3.4) (22) and *panl-3* (ZK632.7) (22).

### Quantification of the *ets-4* 3′-UTR GFP reporter

Images used to quantify the GFP intensity in reporter strains were acquired with an Axio Imager.Z2 (Carl Zeiss, Germany), equipped with an Axiocam 506 mono digital camera (Carl Zeiss, Germany) and a Plan-Apochromat ×63/1.40 Oil DIC M27 objective. Images acquired with the same camera settings were processed with ZEN 2.5 (blue edition) microscope software in an identical manner. The signal intensity of a circular area of 150 pixels in diameter of 5–7 gut nuclei, from five animals per condition, was measured in ImageJ (23) and normalized to the background. In addition, 30–35 animals per strain were visually inspected for GFP expression. Statistical analysis on all of the experiments was performed using GraphPad Prism 6. The statistical methods used to calculate *P*-values are indicated in the figure legends.

### Quantiative real-time polymerase chain reaction (RT-qPCR)

Around 1000 young adult *C. elegans* were collected at 20°C, washed twice in M9 buffer and flash-frozen in Trizol. Total RNA was isolated using the Direct-zol RNA Miniprep Kit (Zymo Research, USA, Cat. No. R2053). A 2000 ng aliquot of RNA was used to prepare cDNA with the High-Capacity cDNA Reverse Transcription Kit (Applied Biosystems, USA, Cat. No. 4368814). cDNA was diluted at 1:10 and 4 μl of template cDNA was mixed with the AMPLIFY ME SG Universal Mix (Blirt, Poland, Cat. No. AM02-200). Ct values were calculated using Light Cycler 480 (Roche, Switzerland). *tbb-2* (β-tubulin) was used as the reference gene. Statistical analysis on all of the experiments was performed using GraphPad Prism 6. The statistical method used to calculate *P*-values is indicated in the figure legends. RT-qPCR primers used in this study are listed in Supplementary Table S2.

### The assay for *C. elegans* cold survival

Cold survival experiments were performed as published (15). Specifically, prior to cold adaptation, animals were grown at 20°C for two generations on OP50. They were then synchronized by bleaching, and L1 larvae were grown until day 1 of adulthood at 20°C. On day 1 of adulthood, they were cold-adapted at 10°C for 2 h and then shifted to 4°C. Animals were sampled at the indicated intervals, and their survival was scored after 24 h recovery at 20°C.

### Oil Red O staining

Oil Red O staining was performed as published (24). A 0.5 g aliquot of Oil Red O was mixed with 100 ml of isopropanol and stirred for 24 h, protected from direct light. This solution was diluted in water to 60%, stirred for 12 h and filtered by a 0.22 μm pore filter. About 3000 young adult animals were washed from the plates, washed three times with M9 and fixed with 75% isopropanol for 15 min with shaking at 1400 rpm. After fixation followed by spinning and removal of isopropanol, animals were suspended in 1 ml of 60% Oil Red O and stained for 3 h on a shaker with maximum speed, covered with aluminum foil. After staining, nematodes were washed four times with phosphate-buffered saline-Tween (PBS-T). Stained animals were placed on 3% agar pads and imaged on a Nikon SMZ25 with DeltaPix color camera with ×60 zoom. All image-processing steps were done with the Fiji/ImageJ software (25). The calculations were made in two steps. The first step was to measure the signal from the red color. After conversion from RGB to HSB color space and background subtraction, red pixels were selected by color thresholding. A binary mask was created with the saturation channel and applied to the thresholded image. After conversion to 32 bits, zero pixel values were replaced by NaN. The integrated density of all remaining pixels was used as an index of the amount of red staining in the animals. In the next step, the area of the nematode was calculated. The image was converted to 8 bits, background subtraction was carried out and a threshold was set to measure the surface of the particles. The signal from red pixels was compared with the nematode's area. Thirty animals were imaged per strain and biological replicate. A two-tailed *t*-test was used to calculate significance with Graph Pad Prism 6.

### Dual-luciferase assay

The 3′-UTRs were cloned into psiCheck-2 plasmid (Promega, USA, Cat. No. C8021) using the XhoI and NotI sites. The short fragment of the *ets-4* 3′-UTR (F1S) was inserted into the AscI site within the *unc-54* 3′-UTR, using Gibson assembly with Gibson assembly Master MIX (NEB, USA, Cat. No. E2611S). Primers used for fragment amplification are listed in Supplementary Table S2. pFLAG-CMV2-Regnase-1 and pFLAG-CMV2-Regnase-1 D141N plasmids were kindly shared by Osamu Takeuchi. REGE-1 cDNA was cloned into pFLAG-CMV2 plasmid using NotI and KpnI restriction sites. HEK293T cells were grown in Dulbecco's modified Eagle’s medium without glucose supplemented with 10% heat-inactivated fetal calf serum. A 10 ng aliquot of pFLAG expression vectors or empty plasmids and 50 ng of psiCheck-2 plasmid were transfected using Effectene transfection reagent (Qiagen, the Netherlands, Cat. No. 301425), following the manufacturer's instructions. Transfection was done in at least three independent biological replicates. The luciferase assay was done using the dual-luciferase reporter assay system (Promega, USA, Cat. No. E1910) following the manufacturer's instructions. For each measurement, an average of two technical replicates was used for further analysis. Statistical analysis on all experiments was performed using GraphPad Prism 6. The statistical methods used to calculate *P*-values are indicated in the figure legends.

### Western blot analysis

Western blot analysis was conducted as described previously (26). Primary antibodies diluted in 5% milk/PBS-T were: mouse anti-actin (1:2000; Merck, Germany, Cat. No. MAB1501), rat anti-MYC (1:1000; Chromotek, Germany, Cat. No. 9e1-20), rat anti-GFP (1:1000; Chromotek, Germany, Cat. No. 3h9-20) and rabbit anti-REGE-1 polyclonal antibody raised against the first 119 amino acids (1:1000; SDIX, USA). Detection was performed with horseradish peroxidase-conjugated secondary antibodies: horse anti-mouse (1:5000; Cell Signaling, USA, Cat. No. 7076S), goat anti-rat (1:5000; Cell Signaling, USA, Cat. No. 7077S) and goat anti-rabbit (1:5000; Cell Signaling, USA, Cat. No. 7074S), radiance ECL detection reagent (Azure Biosystems, Germany, Cat. No. AC2204) and the c600 imaging system (Azure Biosystems, Germany). Western blotting was performed for all the biological replicates. One representative blot is shown in the results.

### RNA co-immunoprecipitation

RNA co-immunoprecipitation was performed on total lysates from *rege-1(rrr13)* or *rege-1(rrr13); rle-1(rrr44)* animals expressing a single-copy RNase-dead REGE-1::GFP transgene. Nematodes were synchronized, grown at 20°C on standard NGM plates and fed with the OP50 *E. coli* until the young adult stage. Pellets were prepared by harvesting nematodes in M9 buffer, washing with harvest buffer (100 mM KCl, 0.1% Triton X-100) and freezing in liquid nitrogen. Lysates from four biological replicates were prepared by grinding the frozen nematode pellets using a mortar and pestle, and dissolving them in the lysis buffer (50 mM HEPES pH 7.5, 150 mM KCl, 5 mM MgCl_2_, 0.1% Triton X-100, 10% glycerol) supplemented with 150 mM phenylmethylsulfonyl fluoride (PMSF), Complete EDTA-free protease inhibitors (Roche, Switzerland, Cat. No. 11873580001), 1 μM pepstatin A, 0.3 μM aprotinin and 200 U of RNase inhibitor (RNasin; Promega, Germany, Cat. No. N2511) at 4°C. Lysates were cleared by centrifugation at 15 000 × *g* for 20 min at 4°C and passed through a 0.45 μm syringe filter. Immunoprecipitations were performed by incubating lysates equivalent to 20 mg of total protein supplemented with RNasin and 1 mM dithiothreitol (DTT) with 80 μl of anti-GFP magnetic beads GFP-Trap (Chromotek, Germany, Cat. No. gtma-20) for 3 h at 4°C while rotating. Protein extract was saved as input for the western blot (1/200), and for RNA isolation (1/20). After immunoprecipitation, beads were washed three times with the lysis buffer. Half of the beads were resuspended in a 2× sodium dodecyl sulfate (SDS) sample loading buffer, boiled for 5 min at 90°C and analyzed by western blot as described above. The other half of the beads were resuspended in 700 μl of Trizol for RNA extraction and frozen in liquid nitrogen. RNAs were extracted using 70% chloroform and precipitated. Then samples were centrifuged, and pellets were washed with 70% ethanol, dried and resuspended in RNase-free water. cDNAs were synthesized using the QuantiTect Reverse Transcription Kit (Qiagen, the Netherlands, Cat. No 205311). RT–qPCR was performed as described above. After RNA extraction and subsequent qPCR analysis, fold enrichment was calculated as previously described (15).

### RNA synthesis and modification

RNAs for RNA pull-down experiment were *in vitro* transcribed using the MEGAshortscript kit (Thermo Fisher Scientific, USA, Cat. No. AM1354) according to the manufacturer's protocol. The DNA templates for transcription were obtained by PCR amplification of the psiCheck-2 vectors using a forward primer containing an SP6 promoter sequence by Phusion HF polymerase (NEB, USA, Cat. No. M0530S). After transcription, RNAs were purified using the RNeasy MinElute Cleanup Kit (Qiagen, the Netherlands, Cat. No. 74204) according to the manufacturer's protocol. RNA purity was confirmed on an agarose gel in denaturing conditions. RNAs (50 pmol) were biotinylated using the Pierce RNA 3′ End Biotinylation Kit (Thermo Fisher Scientific, USA, Cat. No. 20160) according to the manufacturer's protocol, extracted by chloroform:isoamyl alcohol, precipitated and resuspended in RNase-free water.

RNAs for the SHAPE (selective 2′-hydroxyl acylation analyzed by primer extension) experiment were *in vitro* transcribed using MAXIscript SP6 (Thermo Fisher Scientific, USA, Cat. No. AM1310M) according to the manufacturer's protocol. Templates for *in vitro* transcription were obtained by PCR amplification of *ets-4* 3′-UTR variants from the *pDONR P2R–P3 ets-4 3’UTR F1′* plasmid using a forward primer containing an SP6 promoter sequence. RNAs were DNase I treated and recovered by ethanol precipitation. For RNA modification, 20 pmol of RNA were refolded in 20 μl of buffer containing 10 mM Tris–HCl, pH 7.5, 100 mM KCl and 0.1 mM EDTA by heating for 3 min at 95°C, slow cooling to 4°C, adding 100 μl of H_2_O and 50 μl of 5× folding buffer (1×: 40 mM Tris–HCl, pH 7.5, 140 mM KCl, 0.2 mM EDTA, 5 mM MgCl_2_) and incubating for 10 min at 37°C. Folded RNA was divided equally into two tubes and treated with either 8 μl of 100 mM 1M7 in dimethylsulfoxide [DMSO; reaction (+)] or DMSO alone [control (–)] and allowed to react for 5 min at 37°C. RNA was recovered using the Direct‐zol RNA MiniPrep Kit (Zymo Research, USA, Cat. No. R2050) and resuspended in 25 μl of H_2_O.

### *In vitro* translation

DNA templates for *in vitro* translation were obtained by cloning RLE-1, RLE-1 ROQ mutant, RLE-1 *sanroque* mutant or REGE-1 RNase-dead mutant cDNAs (with an N-terminal FLAG tag and a C-terminal MYC tag) into pCS2+ plasmid using ClaI and XbaI restriction sites and the primers listed in Supplementary Table S2. Proteins were *in vitro* translated using the TnT Quick Coupled Transcription/Translation System (Promega, USA, Cat. No. L1170) according to the manufacturer's protocol.

### RNA pull-down

BioMag Nuclease-Free Streptavidin particles (Bangs Laboratories, USA, Cat. No. BM568) were washed and equilibrated with the binding buffer (5 mM HEPES pH 7.5, 70 mM KCl, 20 mM MgCl_2_, 1 mM DTT, 0.1% Triton X-100, 1% glycerol, 20 μg/ml yeast tRNA). Biotinylated RNAs (7 pmol) were incubated with streptavidin beads in the binding buffer supplemented with 80 U of RNasin (Promega, Germany, Cat. No. N2511) and 50 μg of yeast tRNA (Thermo Fisher Scientific, USA, AM7119) for 2 h at 4°C with shaking. Unbound RNAs were washed with the binding buffer; beads were resuspended in the binding buffer and incubated with reticulocyte lysates with *in vitro* translated proteins for 2 h at 4°C with shaking. After incubation, streptavidin beads were washed, resuspended in a 2× SDS sample loading buffer, and proteins were separated using SDS–polyacrylamide gel electrophoresis (PAGE). Proteins bound to RNAs were detected based on their size using western blot analysis as described above.

### SHAPE structure probing

Detection of 2′-*O*-adducts was performed using reverse transcription with ^32^P-labeled primers. Briefly, 1 μl of primer (AGGAATATGTTCTACAACGAACAGT) was added to 0.5 pmol of (–) and (+) RNA, and 12 μl of primer–template solutions were incubated at 95°C for 2 min, followed by 60°C for 5 min and 52°C for 2 min. RNA was reverse transcribed at 52°C for 10 min by Superscript III (Thermo Fisher Scientific, USA, 12574026). Samples and sequencing ladders were purified by ethanol precipitation. Primer extension products were separated by denaturing PAGE (8 M urea). The gels were quantitatively analyzed by phosphoimaging using FLA‐5100 phosphoimager (Fujifilm, Japan) with MultiGaugeV 3.0 software.

## RESULTS

### REGE-1 and Regnase-1 are functional homologs

**Figure 1.**
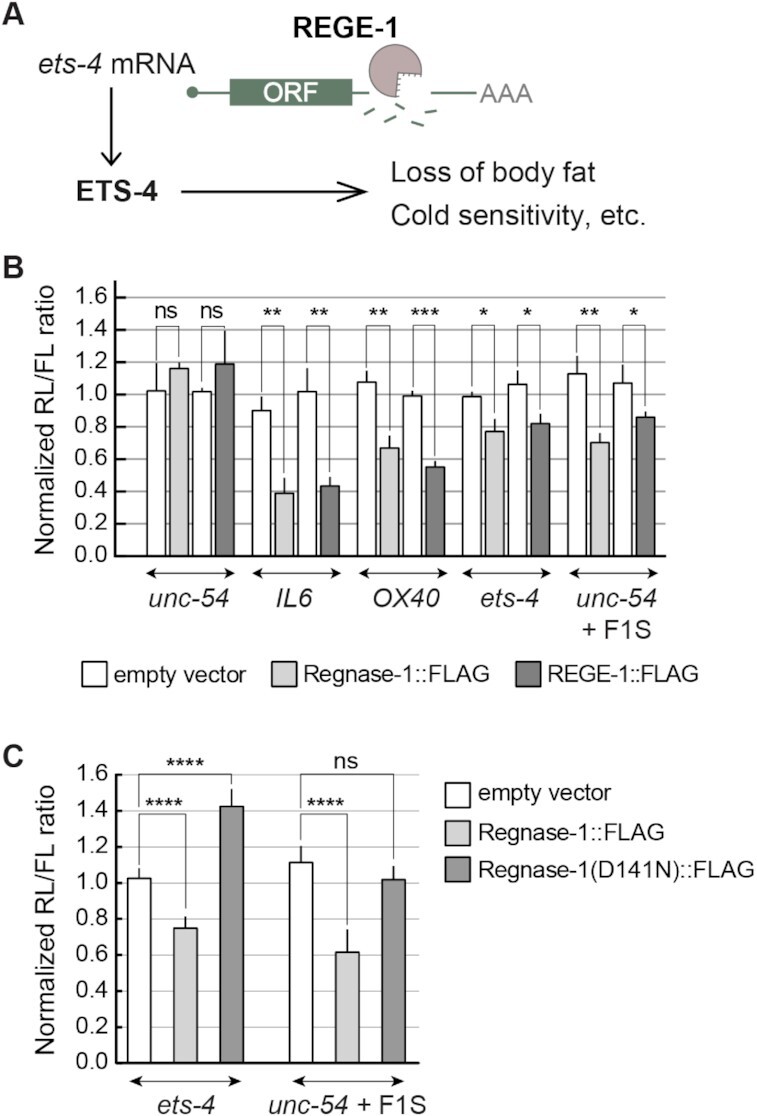
REGE-1 and Regnase-1 are functional homologs. (**A**) A model for REGE-1-mediated control of fat metabolism and cold resistance. By degrading the *ets-4* mRNA, REGE-1 lowers the levels of transcription factor ETS-4. This leads to altered expression of ETS-4 target genes, affecting the accumulation of body fat and cold resistance (15). (**B**) In cells, REGE-1 and Regnase-1 are interchangeable and sufficient for mRNA silencing. HEK cells were transfected with reporters expressed under the control of various 3′-UTRs: from Regnase-1 targets (*OX40* and *IL6*), REGE-1 targets (*ets-4* and *unc-54* + F1S) and an unregulated *C. elegans* mRNA (*unc-54*). Additionally, cells were co-transfected with constructs expressing empty vector (white bars), mouse Regnase-1 (light gray bars) or nematode REGE-1 (dark gray bars). Expression of the *Renilla* luciferase was controlled by the indicated 3′-UTRs and normalized to firefly luciferase expression. Two-tailed *P*-values were calculated with an unpaired Student *t*-test, using empty vector results as the reference. Bars represent the mean value from three independent biological replicates (*n* = 3). Error bars represent the SEM; *P ≤ 0.05, **P ≤ 0.01, ***P ≤ 0.001, ‘ns’ = not significant. (**C**) The RNase activity of Regnase-1 is crucial for the silencing of REGE-1 targets in HEK cells. The same as in (B), except that HEK cells were co-transfected with reporters controlled by 3′-UTRs of REGE-1 targets (*ets-4* and F1S in *unc-54*), and additionally by constructs expressing the empty vector (white bars), mouse Regnase-1 (light gray bars) or its RNase-dead variant (Regnase-1 D141N; dark gray bars). Two-tailed *P*-values were calculated with an unpaired Student *t*-test using empty vector results as the reference. Bars represent the mean value from five independent biological replicates (*n* = 5). Error bars represent the SEM; ****P ≤ 0.0001, ‘ns’ = not significant.

Recently, we characterized the *C. elegans* ortholog of Regnase-1, REGE-1, which affects various aspects of nematode physiology by degrading mRNA encoding the transcription factor ETS-4 (Figure 1A) (15). The *C. elegans* REGE-1 and mammalian Regnase-1 share the same domain organization, suggesting functional conservation (16). To test this, we examined if REGE-1 can degrade mRNA targets of Regnase-1, and vice versa. Specifically, we transfected HEK293T cells with the so-called dual-luciferase (firefly/*Renilla*) reporter constructs, where one reporter is used as a reference and the other as a query. We tested reporters carrying 3′-UTRs of two Regnase-1 targets, *mIL6* (4,6) and *mOX40* (13,27), as well as the 3′-UTR of nematode *unc-54* mRNA that served as a negative control (Figure 1B). Additionally, we examined the silencing potential of the *ets-4* 3′-UTR, which was previously shown to be mediated by a short fragment dubbed F1S. When ‘transplanted’ into an otherwise unregulated *unc-54* 3′-UTR, the F1S fragment is sufficient for REGE-1-mediated silencing (15). We expressed the above reporters in mammalian cells that also expressed either REGE-1, Regnase-1 or an RNase-dead variant of Regnase-1 (Figure 1B and C). We observed that REGE-1 and Regnase-1 were interchangeable. While REGE-1 reduced the expression of reporters known to be targeted by Regnase-1 (*IL6* and *OX40*), Regnase-1 silenced the reporters targeted by REGE-1 (*ets-4* and *unc-54* carrying the F1S fragment) (Figure 1B and C). As expected, the RNase activity of Regnase-1 was crucial for the silencing of REGE-1 targets, as the RNase-dead variant of Regnase-1 (D141N) was unable to induce the silencing (Figure 1C). Intriguingly, we noticed a protective effect of the Regnase-1 D141N mutant on the *ets-4* 3′-UTR reporter (Figure 1C). While it is reminiscent of previous observations on some Regnase-1 targets (6), its significance remains unclear.

### The REGE-1-mediated mRNA silencing requires RLE-1, the *C. elegans* counterpart of Roquin-1

**Figure 2.**
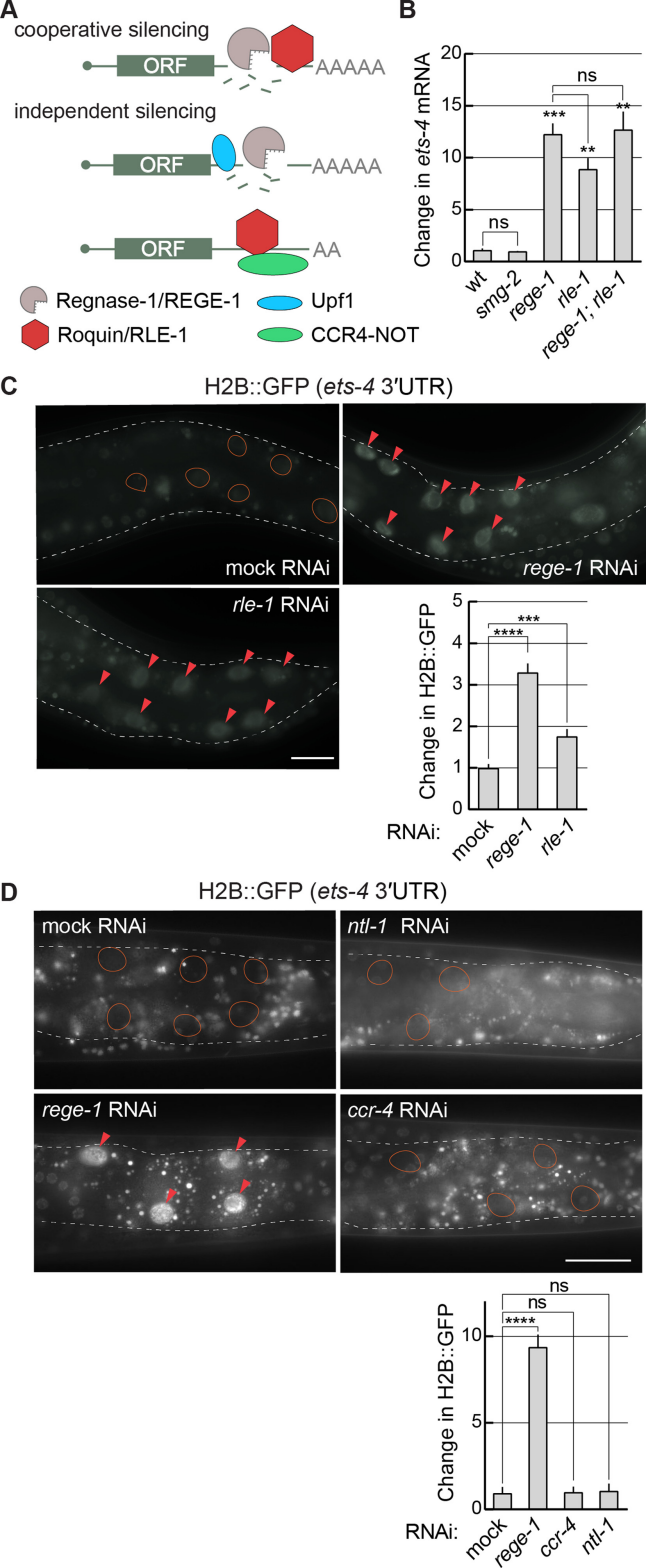
REGE-1 cooperates with RLE-1 in *ets-4* mRNA silencing. (**A**) Two models of mRNA silencing mediated by Regnase-1/REGE-1 and Roquin-1/RLE-1. Based on (4–6). The crucial difference between the two models is in either the cooperative or independent function of two proteins. According to the cooperative model, the RNase activity of Regnase-1 is recruited to specific mRNAs via the interaction with the mRNA-binding Roquin-1. According to the independent model, Regnase-1 and Roquin-1 silence mRNAs independently of each other and they do so by different mechanisms: Regnase-1 regulates translationally active transcripts with the help of Upf1, while Roquin-1 controls translationally inactive mRNAs by recruiting the CCR4–NOT deadenylase complex. (**B**) RLE-1 and REGE-1 are both important for the silencing of *ets-4* mRNA, while SMG-2/Upf1 is not. The levels of *ets-4* mRNA were measured, by RT–qPCR, in animals of the indicated genotypes. Strains used: wild type (wt), *smg-2(rrr60)*, *rege-1(rrr13)*, *rle-1(rrr44)* and *rege-1(rrr13); rle-1(rrr44)* double mutants. The mRNA levels were normalized to the levels of *tbb-2* (tubulin) mRNA. Two-tailed *P*-values were calculated using an unpaired Student *t*-test. Bars represent the mean value from three independent biological replicates (*n* = 3). Error bars represent the SEM; **P ≤ 0.01, ***P ≤ 0.001, ‘ns’ = not significant. (**C**) RLE-1, like REGE-1, silences *ets-4* mRNA via its 3′-UTR. Upper left: partial view of live animals, with outlined intestines, expressing the H2B::GFP reporter from a ubiquitous promoter (*dpy-30*), under the control of the *ets-4* 3′-UTR. The animals were subjected to either mock, *rege-1* or *rle-1* RNAi, as indicated. Ovals mark the positions of gut nuclei not expressing the reporter GFP in control animals. Arrowheads indicate the gut nuclei in which the reporter expression is increased. Scale bar: 20 μm. Lower right: the corresponding quantification of changes in the reporter GFP intensity. Between 5 and 10 nuclei per animal, in at least five animals per condition, were analyzed, to quantify GFP intensities from 30 nuclei in total (*n* = 30). Two-tailed *P*-values were calculated using an unpaired Student *t*-test. Bars represent the mean value from GFP intensity. Error bars represent the SEM. ***P ≤ 0.001, ****P ≤ 0.0001. (**D**) Depletion of the *C. elegans* CCR4–NOT deadenylase has no impact on *ets-4* silencing. Top: partial view of live animals, expressing the H2B::GFP reporter from a ubiquitous promoter (*dpy-30*), under the control of the *ets-4* 3′-UTR. Animals were subjected to either mock, *rege-1* (positive control), *ccr-4* or *ntl-1* (components of the CCR4–NOT complex) RNAi, as indicated. Ovals mark the positions of gut nuclei not expressing the reporter GFP; arrowheads indicate the gut nuclei in which the reporter expression is increased. Scale bar: 50 μm. Bottom: the corresponding quantification of changes in the reporter GFP intensity. Between 5 and 10 nuclei per animal, in at least five animals per condition, were analyzed, to quantify GFP intensities from 30 nuclei in total (*n* = 30). Two-tailed *P*-values were calculated using an unpaired Student *t*-test. Bars represent the mean value from GFP intensity. Error bars represent the SEM. ****P ≤ 0.0001; ‘ns’ = not significant.

Considering the functional similarity between REGE-1 and Regnase-1, we asked whether REGE-1 uses one of the mechanisms proposed for Regnase-1. We thus tested the possible involvement of SMG-2 [the homolog of Upf1, reported to function with Regnase-1 according to the independent model (8)] and RLE-1 (the *C. elegans* counterpart of Roquin-1, functioning either independently or together with Regnase-1) (Figure 2A). Analyzing *smg-2(–)* mutants, we observed the expected increase in the levels of a previously reported target, *yars2b.1* mRNA (28), while the levels of *ets-4* mRNA remained unaffected (Figure 2B and Supplementary Figure S2A). In contrast, the levels of *ets-4* mRNA increased in *rle-1(–)* mutants (Figure 2B); the magnitude of this increase was similar to that observed in either *rege-1(–)* single or *rege-1(–); rle-1(–)* double mutants (Figure 2B). We then tested if RLE-1-dependent silencing of *ets-4* is mediated by the *ets-4* 3′-UTR. To do this, we created a strain expressing histone H2B tagged with GFP (H2B::GFP) under the control of the *ets-4* 3′-UTR; the H2B::GFP fusion concentrates GFP in nuclei, facilitating quantification. Similar to the depletion of *rege-1*, RNAi-mediated depletion of *rle-1* increased this reporter's expression relative to the wild type (Figure 2C). Importantly, the loss of *ets-4* silencing observed in RLE-1-depleted animals was not caused by the loss of REGE-1. Quite the opposite, in RLE-1-depleted animals, we observed increased levels of *rege-1* mRNA and REGE-1 protein (Supplementary Figure S1A), which could be explained by ETS-4-mediated up-regulation of *rege-1* transcription (15). Consistent with this scenario, the up-regulation of *rege-1* mRNA in *rle-1(–)* single mutants was diminished in *rle-1(–); ets-4(–)* double mutants (Supplementary Figure S1B). At the same time, the expression of RLE-1, which is enriched in the intestine (29), was not affected by the depletion of REGE-1 (Supplementary Figure S1C). To summarize, while SMG-2/Upf1 is not involved in REGE-1-mediated silencing, RLE-1 appears to be equally important for the silencing as REGE-1.

When functioning independently of Regnase-1 (according to the independent model), Roquin was shown to induce mRNA turnover by recruiting the CCR4–NOT deadenylase complex (6,30). This is why we additionally examined the potential involvement of the nematode CCR4–NOT complex. Upon RNAi-mediated knock-down of *ntl-1* or *ccr-4*, we observed the expected increases in the levels of a previously reported target, *lip-1* mRNA (20), while the levels of *ets-4* mRNA remained unaffected (Supplementary Figure S2B). Similarly, the depletion of *ntl-1* or *ccr-4* had no impact on the *ets-4* reporter (Figure 2D). Thus, the CCR4–NOT complex appears to be dispensable for the *ets-4* mRNA decay. We additionally RNAi-depleted *pan-2* and *pan-3* (deadenylases functioning independently of CCR4–NOT), or *dcp-1* and *dcp-2* (de-capping enzymes), but observed no effect on the expression of the *ets-4* 3′-UTR reporter (Supplementary Figure S2C). Collectively, our observations suggest that the silencing by REGE-1 requires RLE-1/Roquin, which appears to function independently from mRNA deadenylases and decapping enzymes. Altogether, the functional interdependence between REGE-1 and RLE-1 suggests a mechanism involving collaboration between the two proteins, reminiscent of the model postulating mRNA co-regulation by Regnase-1 and Roquin-1 (4).

### RLE-1 and REGE-1 have overlapping biological functions

**Figure 3.**
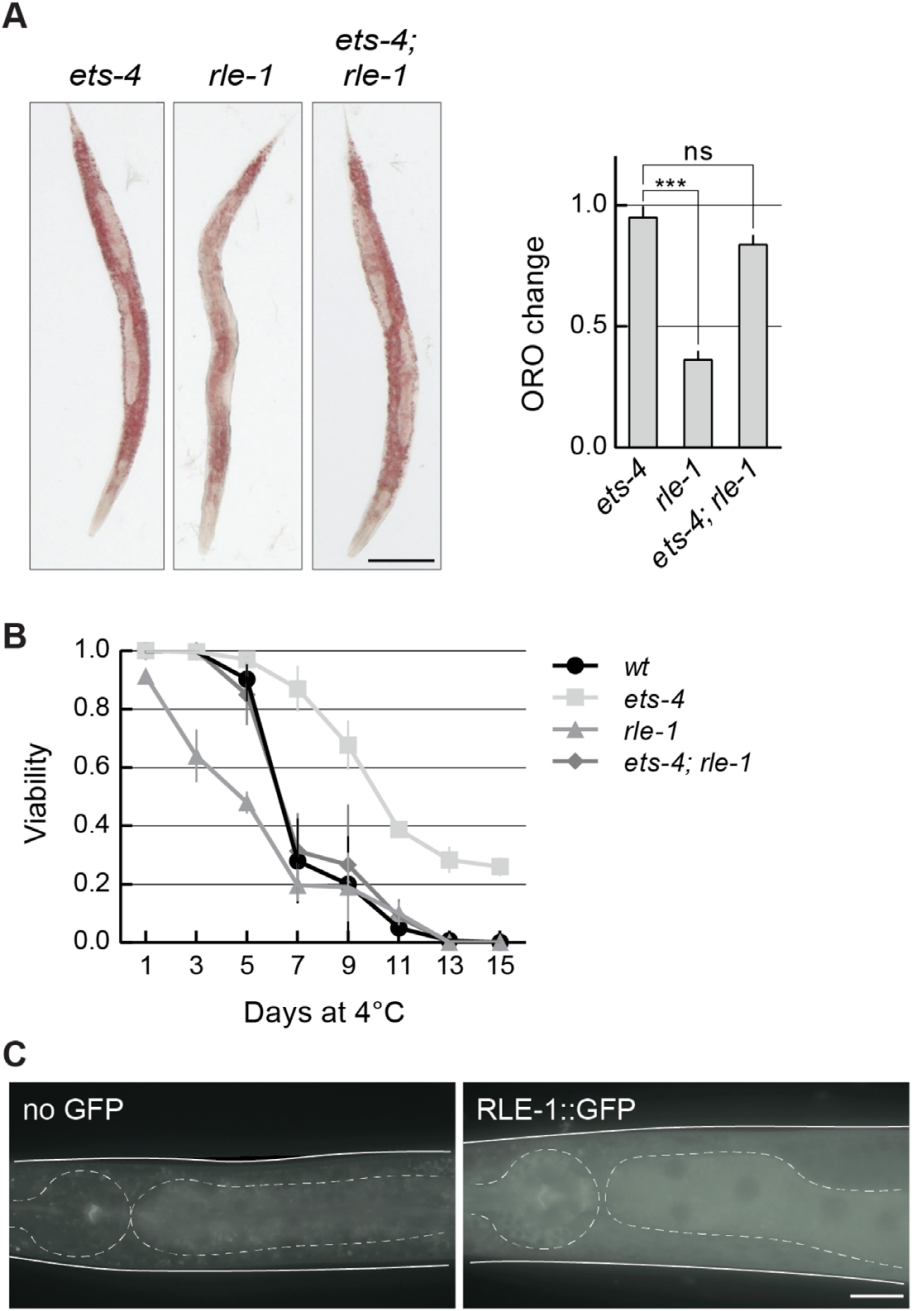
RLE-1 has similar biological roles to REGE-1. (**A**) RLE-1 prevents ETS-4-dependent loss of body fat. Left: representative pictures of animals of the indicated genotypes with body fat stained with the lipophilic dye Oil Red O (ORO). Scale bar: 100 μm. Right: the corresponding quantification of body fat. The experiment was performed in three biological replicates (*n* = 3); 10–15 animals were scored per replicate. Two-tailed *P*-values were calculated using an unpaired Student *t*-test. Bars represent the mean value from all biological replicates. Error bars represent the SEM. ‘ns’ = not significant, ***P ≤ 0.001. (**B**) RLE-1 is important for wild-type cold survival. Animals of the indicated genotypes were exposed to cold for the indicated time, and examined for viability one day after rewarming at 20ºC. Tested were: wt (black circles), *ets-4(rrr16)* (light gray squares)*, rle-1(rrr44)* (gray triangles) and *ets-4(rrr16); rle-1(rrr44)* double mutants (dark gray rhombi). The experiment was performed in three biological replicates (*n* = 3); ∼100 animals were scored per time point. Bars represent the mean value from all replicates. Error bars represent the SEM. (**C**) RLE-1 is a ubiquitous protein enriched in the intestine. Partial view of representative live animals, which either did not (left) or did (right) express the endogenously tagged RLE-1::GFP protein. To reduce gut-specific autofluorescence, the animals carried the *glo-1(zu391)* mutation (34). As a control (no GFP) *glo-1* animals were used. The pharynx and intestines are outlined. Scale bar: 20 μm.

Since both REGE-1 and RLE-1 are required for the silencing of *ets-4* mRNA, one could expect similar biological functions for both proteins. We previously showed that REGE-1 facilitates the accumulation of body fat and cold resistance by lowering the levels of ETS-4 (Figure 1A) (15). Similarly, we observed that *rle-1(–)* mutants had reduced levels of body fat and were more cold sensitive (Figure 3A and B). Moreover, both phenotypes were reversed by the loss of ETS-4 (Figure 3A and B). These results are consistent with the silencing of *ets-4* mRNA by both proteins. Also consistent with the co-regulation, RLE-1 and REGE-1 are both expressed in the intestine (15,29). Nonetheless, RLE-1 was reported to be expressed in additional tissues where REGE-1 is not detectable (29). To confirm this, we created a strain expressing GFP-tagged RLE-1 from the endogenous locus. In agreement with previous studies, we observed a strong expression in the intestine and a weak but ubiquitous expression in non-intestinal tissues (Figure 3C). Thus, in addition to co-silencing the *ets-4* with REGE-1, RLE-1 may play additional roles independent from REGE-1.

### REGE-1 displays a high specificity towards *ets-4* mRNA

**Figure 4.**
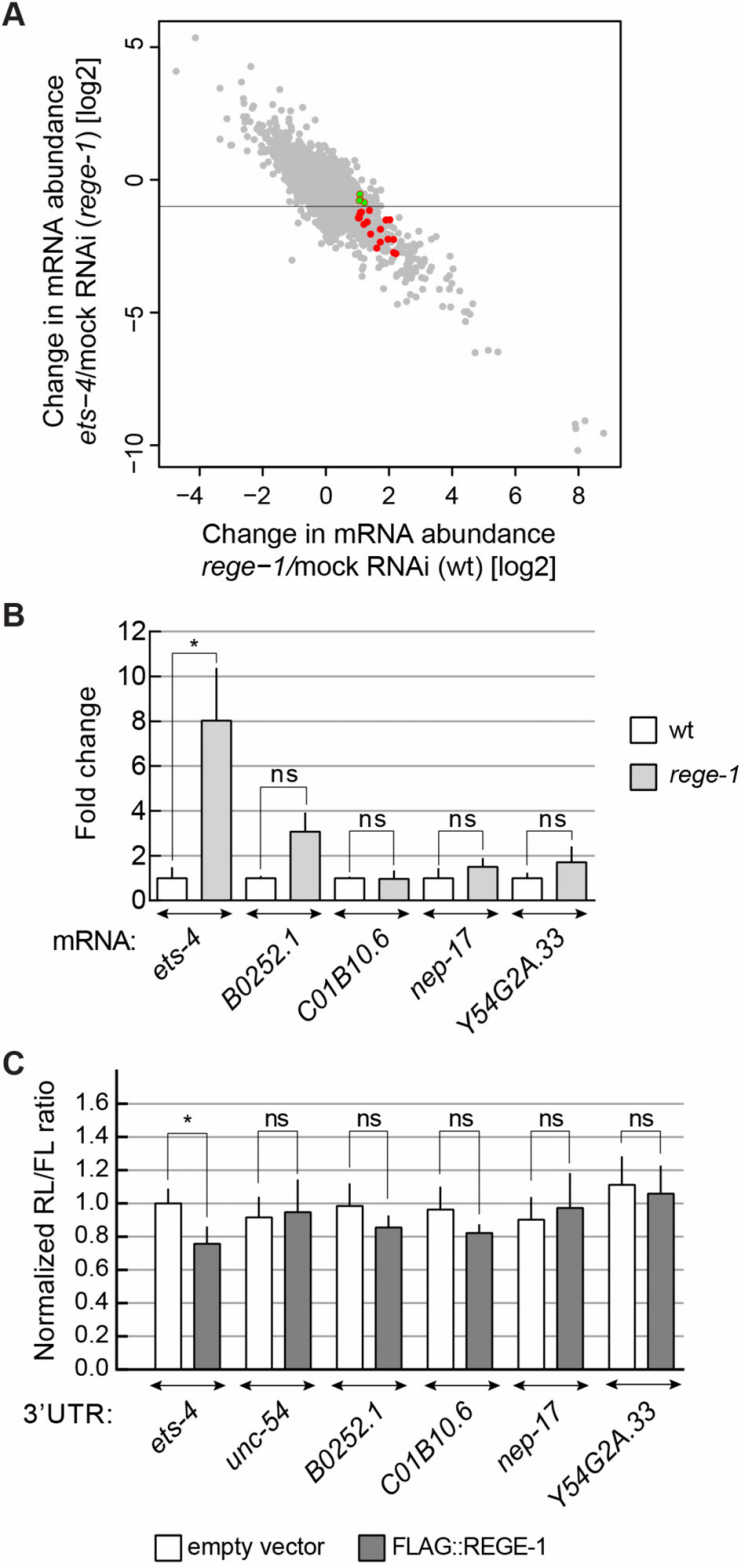
*ets-4* mRNA is the major REGE-1 target. (**A**) Plot comparing changes in gene expression upon *rege-1* RNAi on otherwise wild-type animals (*x*-axis) versus *ets-4* RNAi on *rege-1(rrr13)* mutant animals (*y*-axis); based on published expression data (15). Colored are mRNAs potentially degraded by REGE-1, based on published EISA (15). Note that all these mRNAs change in abundance in *rege-1(rrr13)* mutants upon the depletion of *ets-4*, suggesting that their expression depends on ETS-4. In green are four transcripts analyzed below, whose abundance changed < 2-fold. (**B**) The levels of additional candidate mRNAs are not regulated by REGE-1. The levels of *ets-4* (positive control), *B0252.1*, *C01B10.6*, *nep-17* and *Y54G2A.33* mRNAs were measured, by RT–qPCR, in animals of the indicated genotypes. Strains used: wt and *rege-1(rrr13)*. The levels were normalized to *tbb-2* (tubulin) mRNA. Bars represent the mean from four biological replicates (*n* = 4), error bars represent the SEM. Two-tailed *P*-values were calculated using unpaired Student *t*-test compared with the wt. ‘n.d.’ = not detected, ‘ns’ = not significant, *P ≤ 0.05. (**C**) The 3′-UTRs of selected candidates do not direct REGE-1-mediated silencing in HEK cells. HEK cells were transfected with reporters expressed under the control of various 3′-UTRs: from *ets-4* (positive control)*, B0252.1*, *C01B10.6*, *nep-17* and *Y54G2A.33* mRNAs. Additionally, cells were co-transfected with constructs expressing empty vector (white bars) or REGE-1 (gray bars). Expression of the *Renilla* luciferase was controlled by the indicated 3′-UTRs and normalized to firefly luciferase expression. Two-tailed *P*-values were calculated with an unpaired Student *t*-test, using empty vector results as the reference. Bars represent the mean value from four independent biological replicates (*n* = 4). Error bars represent the SEM; ‘ns’ = not significant, *P ≤ 0.05.

Our studies suggest that ETS-4 is responsible for most, if not all, changes in gene expression and phenotypes associated with the loss of REGE-1 [(15) and this study]. Thus, it is not clear why/whether additional mRNAs are degraded by REGE-1. To test this further, we re-examined the data that led to the identification of *ets-4* mRNA as a REGE-1 target. Previously, exon–intron split analysis (EISA) identified a small group of mRNAs whose abundance in *rege-1* mutants appeared to increase compared with the wild type, preferentially at the level of mature but not nascent transcript (15). The *ets-4* mRNA was the best candidate, and subsequent functional analysis supported the regulation of this but not of additional candidates by REGE-1 (15). Nonetheless, some mRNAs could be degraded by REGE-1 without obvious phenotypic consequences, which is why we examined several additional candidates from the EISA. We reasoned that the abundance of a bona fide REGE-1 target should increase in REGE-1-deficient animals, but not be affected by the additional depletion of ETS-4. Using these criteria, all previously identified candidates are affected by the depletion of ETS-4, arguing that their up-regulation in *rege-1(–)* animals is indirectly caused by the increased expression of ETS-4 (Figure 4A). Nonetheless, we examined further four mRNAs: *B0252.1*, *C01B10.6*, *nep-17* and *Y54G2A.33*, whose levels appeared to be the least dependent on ETS-4 [their abundance changed < 2-fold in *rege-1(–)* mutants upon *ets-4* RNAi; green transcripts in Figure 4A]. The F1S element of the *ets-4* 3′-UTR is highly conserved between nematodes (15). However, we did not observe such conservation within the 3′-UTRs of candidate mRNAs. Still, we decided to test if the stability of candidate targets is affected by REGE-1 *in vivo* and re-examined their abundance in *the rege-1(–)* mutant. In contrast to the *ets-4* mRNA, none of the tested transcripts was affected by the loss of REGE-1 (Figure 4B). Finally, we used the dual-luciferase assay in mammalian cells (like in Figure 1B) to test the silencing potential of 3′-UTRs from the selected candidates. In contrast to the *ets-4* 3′-UTR, none of the candidate 3′-UTRs had an impact on the reporter expression (Figure 4C). Thus, all data so far suggest that *ets-4* is the main, if not exclusive, target of REGE-1-mediated regulation.

### The *ets-4* 3′-UTR contains RNA stem–loops, ADE and RCE, which are required for its silencing

**Figure 5.**
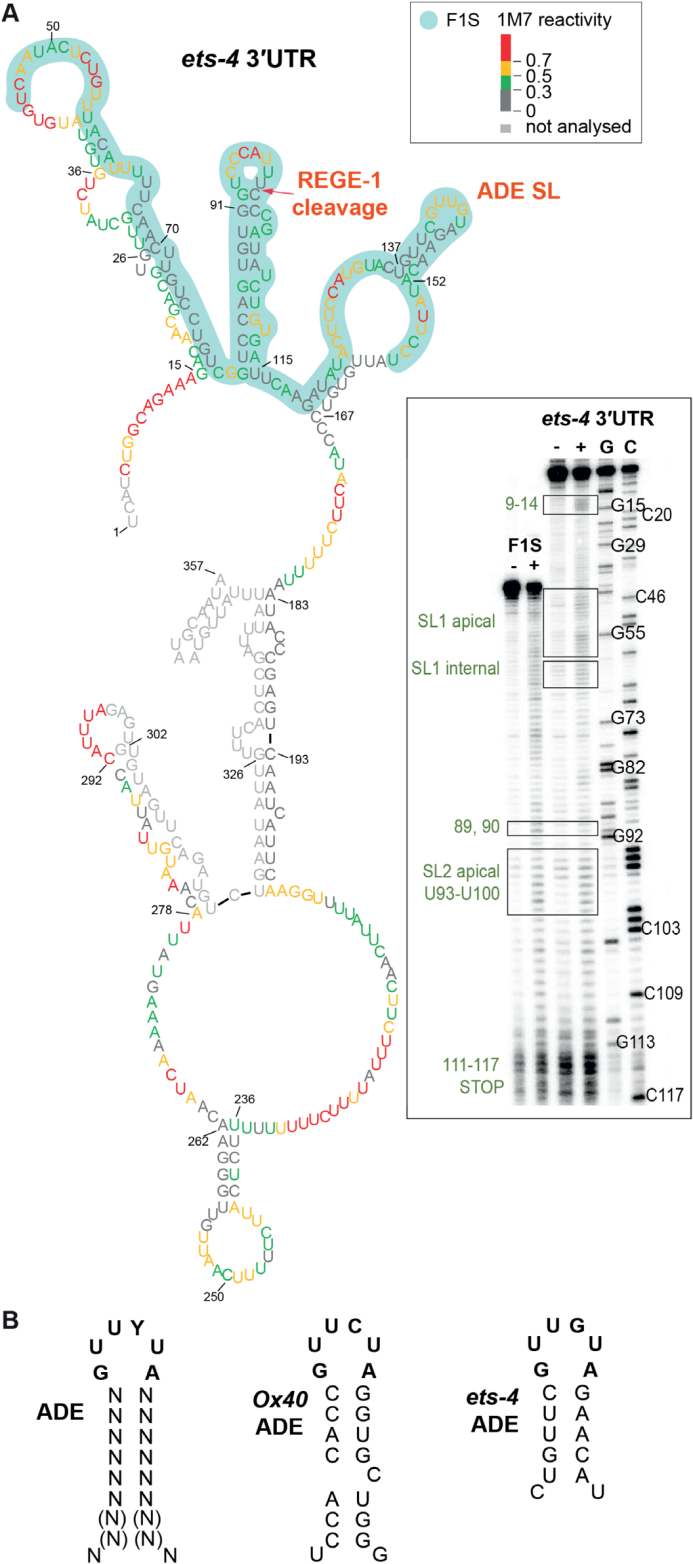
The F1S fragment of the *ets-4* 3′-UTR contains the ADE-type SL, potentially mediating the regulation by RLE-1. (**A**) The F1S fragment of the *ets-4* 3′-UTR contains an ADE SL. Shown is the *ets-4* 3′-UTR structure obtained by SHAPE *in vitro* probing. The RNA reactivity patterns (labeled as 1M7 reactivity) were obtained for the isolated F1S sequence, or in the context of the whole *ets‐4* 3′-UTR. The F1S encompasses a 115 nt fragment, between residues U45 and U159 (highlighted in light green). The red arrow indicates the REGE-1 cleavage site, based on (15). The consensus motif in the ADE SL is indicated as ADE SL. Gel electrophoresis fractionation of products resulting from SHAPE structure probing is shown on the bottom right. Both RNAs (F1S and *ets-4*) were run concurrently to allow direct comparison. Lane (−) represents the control sample with untreated RNA; lane (+) 1M7 modification, C and G are sequencing lanes. (**B**) The ADE SL from the *ets-4* 3′-UTR is nearly identical to the motif recognized by Roquin-1. Left: mammalian consensus for ADE SL recognized by mammalian Roquin-1, based on (12). Middle: ADE-type SL from mammalian mRNA *OX40*. Right: ADE-like SL from *ets-4* mRNA.

**Figure 6.**
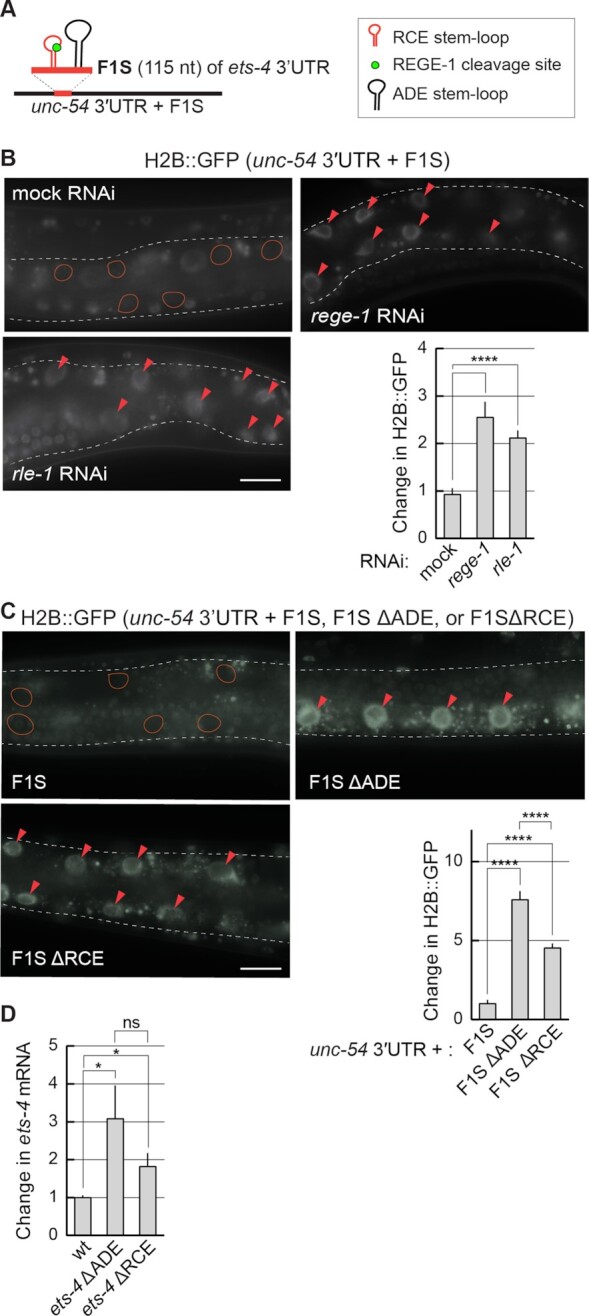
The F1S fragment is sufficient for REGE-1- and RLE-1-mediated mRNA regulation. (**A**) Schematic representation of a hybrid 3′-UTR, controlling the expression of the GFP reporter. The F1S fragment from the *ets-4* 3′-UTR, which mediates REGE-1-induced degradation, was ‘transplanted’ into an otherwise unregulated 3′-UTR, resulting in a ‘hybrid’ 3′-UTR (*unc-54* 3′UTR + F1S). The F1S was inserted between base pairs 164 and 165 of the *unc-54* 3′-UTR; the F1S contains the REGE-1 cleavage site within the RCE SL, next to the ADE SL. (**B**) The silencing mediated by the F1S fragment requires both REGE-1 and RLE-1. Upper left: partial view of live, wild-type animals, with outlined intestines, expressing the H2B::GFP reporter under the control of a modified *unc-54* 3′-UTR (containing the F1S fragment of *ets-4*, indicated as the *unc-54* 3′UTR + F1S). The animals were subjected to either mock, *rege-1* or *rle-1* RNAi, as indicated. Ovals mark positions of gut nuclei in which, in the wild type, the reporter is silenced. Arrowheads indicate the gut nuclei expressing the reporter GFP. Scale bar: 20 μm. Bottom right: the corresponding quantification of changes in the GFP intensity. Between 5 and 10 nuclei per animal, in at least five animals per condition, were analyzed to obtain GFP intensities from 35 nuclei in total (n = 35). Two-tailed P-values were calculated using an unpaired Student t-test. Bars represent the mean value from GFP intensity. Error bars represent the SEM. ****P ≤ 0.0001. (**C**) The deletions of either ADE or RCE result in the loss of reporter silencing. Upper left: partial view of live, wild-type animals, with outlined intestines, expressing the H2B::GFP reporter under the control of the modified *unc-54* 3′-UTR, containing the F1S fragment of the *ets-4* 3′-UTR or its versions with deleted ADE (ΔADE) or RCE (ΔRCE) SLs. Ovals demarcate the gut nuclei in which, in the wild type, the reporter expression is silenced. Arrowheads indicate the gut nuclei expressing the reporter GFP. Scale bar: 20 μm. Lower right: the corresponding quantification of changes in the GFP intensity. Between 5 and 10 nuclei per animal, in at least five animals per condition, were analyzed to quantify 35 nuclei in total (*n* = 35). Error bars represent the SEM. An unpaired two-tailed *t*-test was used to calculate the *P*-value. The *unc-54*3′-UTR + F1S (F1S for short) was used as a reference. ****P ≤ 0.0001. (**D**) ADE and RCE SLs are important for the silencing of *ets-4* mRNA *in vivo*. The level of *ets-4* mRNA was measured, by RT–qPCR, in animals of the indicated genotypes. Strains used: wt, *ets-4(rrr63)*—expressing *ets-4* with the ADE SL deleted, and *ets-4(rrr64)—*expressing *ets-4* with the RCE SL deleted. The mRNA levels were normalized to the levels of *tbb-2* (tubulin) mRNA. Two-tailed *P*-values were calculated using an unpaired Student *t*-test. Bars represent the mean value from six independent biological replicates (*n* = 6). Error bars represent the SEM; *P ≤ 0.05, ‘ns’ = not significant.

The degradation of *ets-4* mRNA by REGE-1 is mediated by a 115 nucleotide long fragment of the *ets-4* 3′-UTR (the F1S fragment) (15). Roquin-1 is thought to bind RNA primarily via its ROQ domain (also present in RLE-1), which recognizes RNA SLs (10). Among them are the so-called ADE SLs containing six nucleotides in the loop (12,13). By scanning the F1S sequence for putative regulatory motifs, we found an ADE-type SL, whose structural organization was experimentally confirmed by SHAPE (Figure 5A). The sequence of this SL fits the ADE consensus recognized by the ROQ domain of Roquin-1 (12), and is almost identical to the ADE SL found in the *OX40* 3′-UTR (Figure 5B) (11,13). Intriguingly, the *ets-4* ADE SL is found next to another SL, which we called the RCE (REGE-1 cleavage element), containing the REGE-1 cleavage site (Figure 5A) (15). Thus, RLE-1 could induce *ets-4* turnover via the association with the ADE SL within the F1S fragment of the *ets-4* 3′-UTR. Indeed, we found that the regulation of GFP reporter expression (mediated by the F1S fragment of the *ets-4* 3′-UTR inserted into the otherwise unregulated *unc-54* 3′-UTR) required both REGE-1 and RLE-1 (Figure 6A and B). These results suggest that the F1S fragment contains all the information necessary for the silencing by both proteins. We then asked whether ADE and RCE are important for the silencing. To test this, we created versions of the F1S GFP reporter carrying deletions of either ADE (F1S ΔADE) or RCE (F1S ΔRCE). Monitoring the expression of these reporters, we found that the deletion of either SL resulted in the loss of silencing (Figure 6C). Additionally, we tested if these SLs are necessary to reduce the levels of endogenous *ets-4* mRNA. To examine this, we generated strains carrying deletions of individual SLs by CRISPR/Cas9 gene editing. Indeed, deleting each SL resulted in elevated levels of the endogenous *ets-4* mRNA compared with the wild type (Figure 6D). Collectively, our data suggest that both ADE and RCE are necessary for the silencing of *ets-4* mRNA.

### RLE-1 binds the ADE-like SL via its ROQ domain

**Figure 7.**
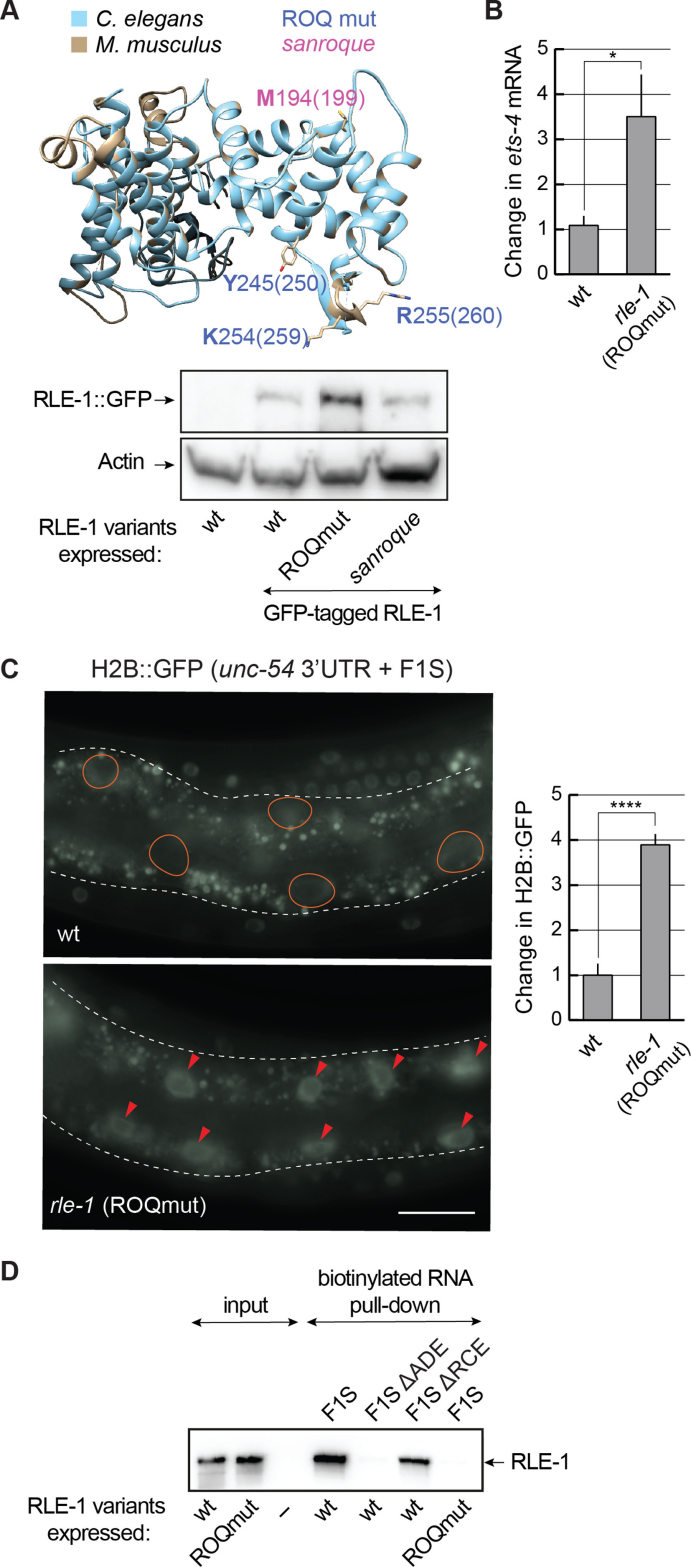
The ROQ domain of RLE-1 is essential for *ets-4* silencing. (**A**) The ROQ domain is conserved between RLE-1 and Roquin-1. Top: a homology model of the *C. elegans* RLE-1 ROQ domain. The RLE-1 ROQ domain tertiary structure was predicted based on conservation with the ROQ domain of mouse Roquin-1 (31), using Phyre2 software. The RLE-1 ROQ domain is in light blue and the Roquin-1 ROQ domain is in gray. Amino acids mutated in the ROQmut (Y245, K254 and R255) (marked in blue) and *sanroque* mutant (M194) (marked in magenta) are indicated. Bottom: western blot of lysates from wild-type (wt) or mutant animals; *rle-1(rrr58)—*expressing GFP-tagged RLE-1, *rle-1(rrr59)—*expressing GFP-tagged RLE-1 ROQmut and *rle-1(rrr62)*—expressing GFP-tagged RLE-1 *sanroque* mutant, detected with the GFP antibody. The experiment was performed twice (*n* = 2). (**B**) Mutations in the ROQ domain (Y245A, K254A and R255A) result in elevated levels of *ets-4* mRNA. The levels of endogenous *ets-4* mRNA were measured, by RT–qPCR, in animals of the indicated genotypes. Strains used: wt, *rle-1*(*rrr61)*—expressing the RLE-1 ROQmut. Bars represent the mean from five biological replicates (*n* = 5), error bars represent the SEM. Two-tailed *P*-values were calculated using unpaired Student *t*-test, the wt was used as a reference. *P ≤ 0.05. (**C**) Mutations in the RLE-1 ROQ domain impair the F1S reporter silencing. Left: partial view of live animals, either wt or expressing RLE-1 with substitutions in the ROQ domain (ROQmut: Y245A, K254A and R255A) (as in B), and expressing the H2B::GFP reporter under the control of a modified *unc-54* 3′-UTR (*unc-54*+ F1S). The intestines are outlined, ovals demarcate the gut nuclei in which, in the wt, the reporter expression is silenced, arrowheads indicate the gut nuclei expressing the reporter GFP. Scale bar: 20 μm. Right: the corresponding quantification of changes in the GFP intensity. Between 5 and 10 nuclei per animal, in at least five animals per condition, were analyzed, to quantify GFP intensities from 35 nuclei in total (*n* = 35). Two-tailed *P*-values were calculated using an unpaired Student *t*-test, the wt was used as a reference. Bars represent the mean value from GFP intensity. Error bars represent the SEM. ****P ≤ 0.0001. (**D**) The ADE SL mediates the RNA binding of RLE-1. *In vitro* synthesized, biotinylated RNAs (F1S, F1S ΔADE or F1S ΔRCE) were purified from cell lysates following the incubation with *in vitro* translated wt RLE-1, or the RLE-1 mutant (ROQmut) carrying amino acid substitutions (Y245A, K254A and R255A) in the ROQ domain. Proteins associated with the biotinylated RNAs were examined by a western blot. The experiment was performed three times (*n* = 3).

Since the ROQ domain is conserved between RLE-1 and Roquin-1, and binds the ADE SLs (Figure 7A) (11–13), we wondered whether this domain also mediates the binding of RLE to the F1S ADE. To examine this, we created a strain expressing a mutant form of RLE-1. By editing the endogenous *rle-1* locus, we introduced mutations resulting in amino acid substitutions (Y245A, K254A and R255A) in the ROQ domain (RLE-1 ROQmut); these correspond to the substitutions in Roquin-1 (Y250A, K259A and R260A) that abolish the RNA binding (13,31). While these mutations did not decrease the abundance of RLE-1 ROQmut protein (Figure 7A), they affected the abundance of the endogenous *ets-4* mRNA and the silencing of the F1S reporter (Figure 7B and C). Then, to examine the interaction between the RLE-1 ROQ domain and the *ets-4* ADE, we attempted to immunoprecipitate RLE-1 but, possibly due to low protein levels, we were unsuccessful. We thus synthesized RLE-1 (or its ROQmut variant) in reticulocyte lysates and performed RNA pull-downs using as a bait biotinylated F1S or its mutant versions. The association with RLE-1 variants was monitored by western blotting. Using this approach, we did observe the interaction between RLE-1 and the F1S RNA. Importantly, the interaction was lost when the F1S ADE (but not RCE) was deleted, or when the RLE-1 ROQmut protein was used instead of wild-type protein (Figure 7D). These results suggest that RLE-1 binds the *ets-4* 3′-UTR via the ADE SL. Also, at least *in vitro*, that binding is independent of RCE and REGE-1.

### REGE-1’s association with *ets-4* is independent of RLE-1

**Figure 8.**
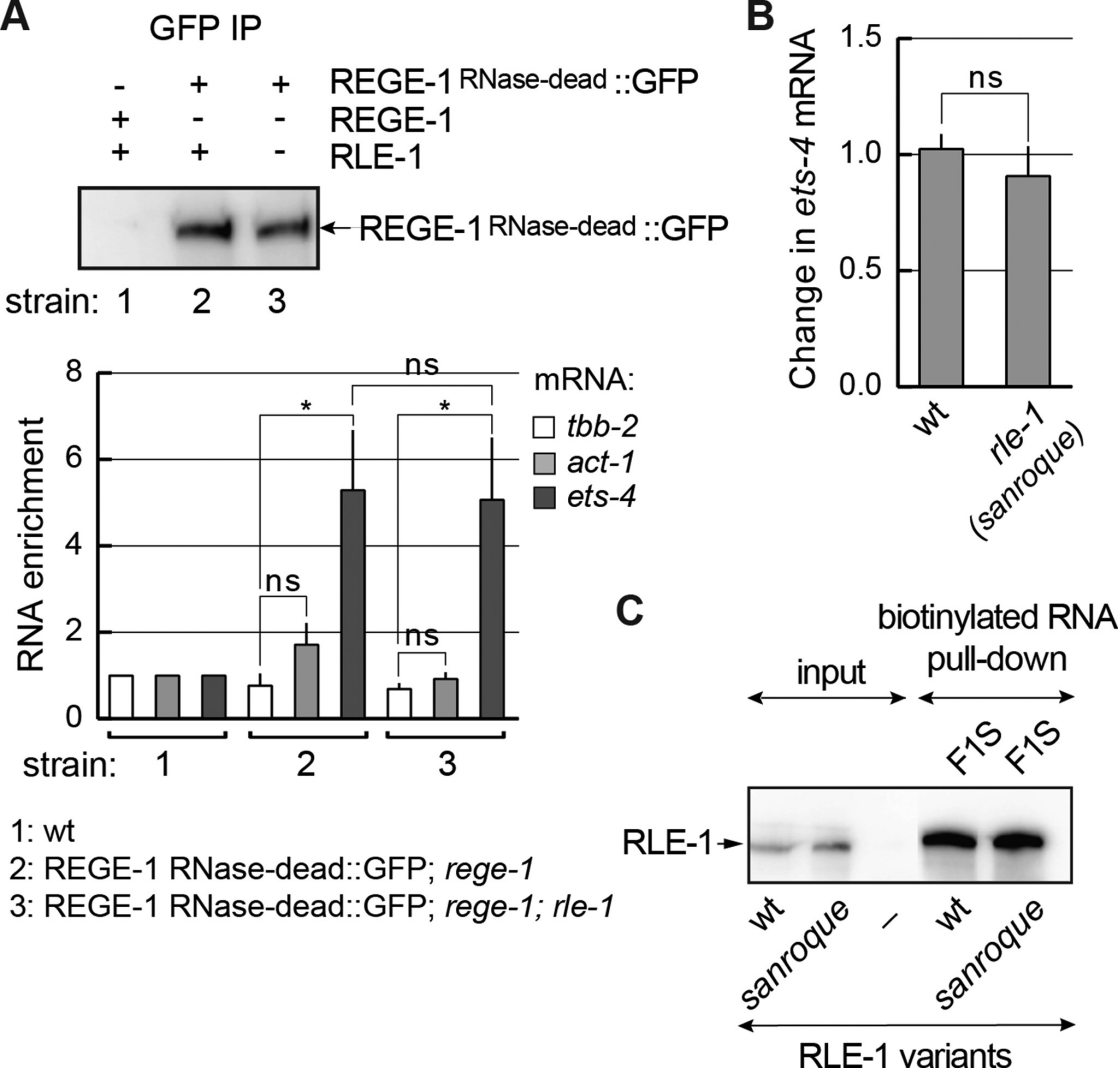
REGE-1 association with *ets-4* is independent of RLE-1. (**A**) REGE-1 associates with the *ets-4* mRNA independently of RLE-1. Animals of the indicated genotypes were lysed and the extracts were subjected to immunoprecipitation (IP), using anti-GFP antibodies. Top: western blot of immunoprecipitates from the wt (strain 1) or mutants; *rege-1(rrr13)* (strain 2) or *rege-1(rrr13); rle-1(rrr44)* double mutants (strain 3) expressing the RNase-dead REGE-1::GFP, detected with REGE-1 antibody (15). Below: the corresponding quantification of the indicated mRNAs, by RT-qPCR, which co-precipitated with the RNase-dead REGE-1. Note that the RNase-dead REGE-1::GFP is associated with *ets-4* mRNA independently of RLE-1. The mRNA levels were normalized to the levels of *tbb-2* (tubulin) mRNA; *act-1* (actin) mRNA was included as a negative control. Bars represent the mean from four biological replicates (*n* = 4), error bars represent the SEM. Two-tailed *P*-values were calculated using unpaired Student *t*-test; lines above the bars indicate values that were compared; ‘ns’ = not significant, *P ≤ 0.05. (**B**) The *sanroque* mutation has no effect on the *ets-4* mRNA silencing. The levels of endogenous *ets-4* mRNA were measured, by RT–qPCR, in animals of the indicated genotypes. Strains used: wt, *rle-1(rrr62)*—expressing the GFP-tagged RLE-1 *sanroque* mutant. Bars represent the mean from five biological replicates (*n* = 5), error bars represent the SEM. Two-tailed *P*-values were calculated using unpaired Student *t*-test, the wt was used as a reference. ‘ns’ = not significant. (**C**) *In vitro* synthesized, biotinylated F1S RNA was purified from cell lysates following incubation with *in vitro* translated wt RLE-1, or the RLE-1 *sanroque* mutant. Proteins associated with biotinylated RNAs were examined with a western blot. The experiment was performed twice (*n* = 2).

We attempted to use the above *in vitro* approach to test also the interaction between F1S and REGE-1. However, REGE-1 associated with the biotinylated F1S non-specifically (in the presence or absence of RLE-1, data not shown). However, *in vivo*, we previously demonstrated a specific association between *ets-4* mRNA and RNase-dead REGE-1 (15). Thus, to test if this association depends on RLE-1, we examined it in *rle-1(–)* mutants. Importantly, we observed that REGE-1 was able to associate with *ets-4* mRNA in both the presence and absence of RLE-1 (Figure 8A). According to a recent study, Regnase-1 can be recruited to mRNA via a physical association with Roquin-1, providing this protein complex with RNA binding specificity. The association between Regnase-1 and Roquin-1 is impaired by a specific mutation in Roquin's ROQ domain, the M199R amino acid substitution also known as the *sanroque* mutation (5). Although REGE-1 appears to associate with *ets-4* independently from RLE-1 (Figure 8A), we edited the endogenous *rle-1* gene to create a *sanroque*-like allele, encoding the RLE-1 variant with the M194R substitution corresponding to M199R in Roquin (Figure 7A). We observed that, *in vivo*, this mutation had no impact on either the abundance of this RLE-1 variant (Figure 7A) or the silencing of *ets-4* mRNA (Figure 8B). Moreover, consistent with unperturbed *ets-4* degradation, the M194R substitution had no impact on the association with biotinylated F1S RNA *in vitro* (Figure 8C). Taken together, our data support an independent association of REGE-1 and RLE-1 with *ets-4* mRNA.

### RLE-1, ADE and RCE SLs are all important for the *ets-4* decay

**Figure 9.**
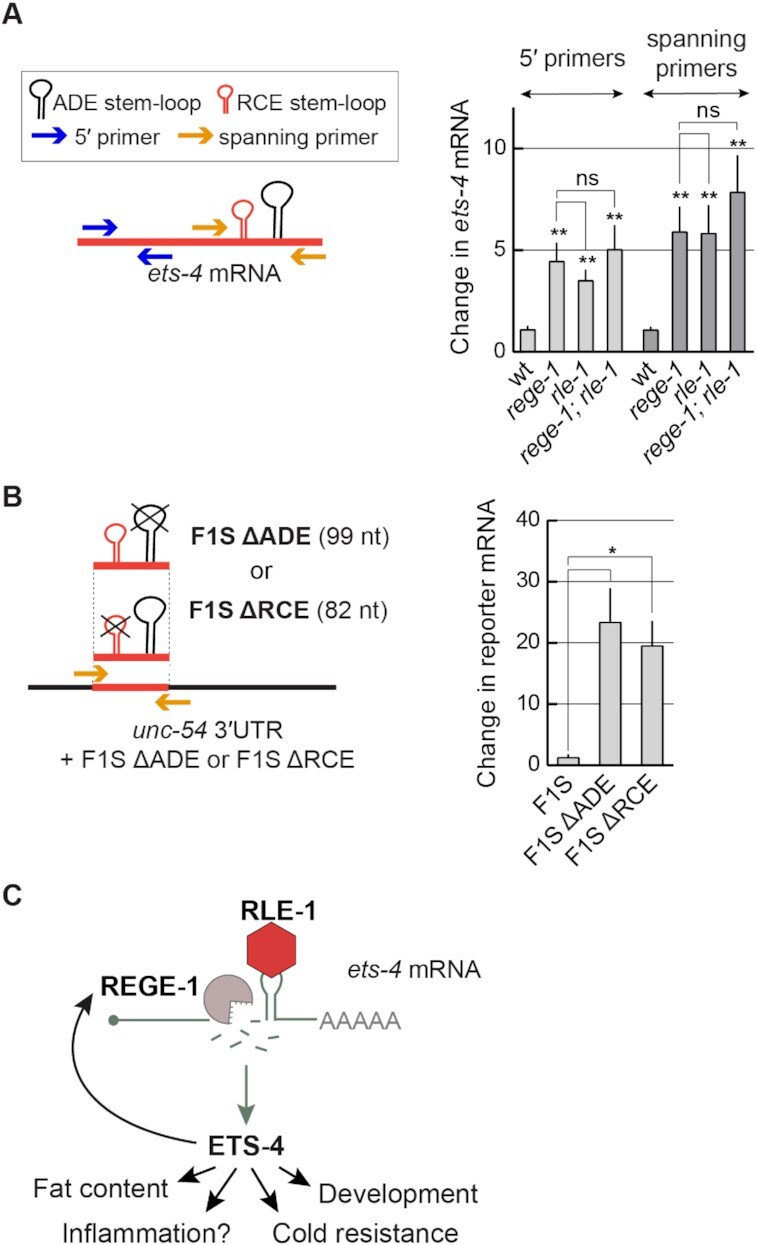
RLE-1 is crucial for REGE-1-mediated RNA cleavage. (**A**) The *ets-4* mRNA fragments spanning the REGE-1 cleavage site are as abundant in the absence of REGE-1 as in the absence of RLE-1. Left: schematic representation of the *ets-4* 3′-UTR and RT–qPCR primers designed to amplify fragments upstream (blue arrows) or spanning (orange arrows) the REGE-1 cleavage site. Right: the levels of *ets-4* mRNA were measured, by RT–qPCR, in animals of the indicated genotypes. Strains used: wt, *rege-1(rrr13)*, *rle-1(rrr44)* and *rege-1(rrr13); rle-1(rrr44)* double mutants. The mRNA levels were normalized to the levels of *tbb-2* (tubulin) mRNA. Bars represent the mean from six biological replicates (*n* = 6), error bars represent the SEM. Two-tailed *P*-values were calculated using unpaired Student *t*-test compared with the wt. ‘ns’ = not significant, **P ≤ 0.01. (**B**) The F1S reporter lacking either the ADE or the RCE SL is no longer degraded. Left: schematic representation of the *unc-54* 3′-UTR + F1S ΔADE or F1S ΔRCE, and the RT–qPCR primers designed to amplify fragment spanning the REGE-1 cleavage site (orange arrows). Right: the levels of F1S, F1S ΔADE and F1S ΔRCE reporter mRNAs were measured, by RT–qPCR. Bars represent the mean from three biological replicates (*n* = 3), error bars represent the SEM. Two-tailed *P*-values were calculated using unpaired Student *t*-test; lines above the bars indicate values that were compared. Error bars represent the SEM, *P ≤ 0.05. (**C**) The proposed model for REGE-1 and RLE-1 (R2)-mediated control of gene expression. Each RBP binds the *ets-4* mRNA independently but the binding of both proteins is crucial for its decay. According to this model, ADE-recruited RLE-1 is essential for mRNA-associated REGE-1 to cleave the mRNA, leading to its destruction. Whether either protein binds to mRNA alone or in association with other proteins remains to be determined. The *ets-4* mRNA seems to be the major REGE-1 target, as the expression of ETS-4 explains essentially all changes in gene expression (including up-regulation of *rege-1* transcription) and phenotypes observed in *rege-1(–)* mutants (15).

Our data indicate that RCE and ADE, as well as RLE-1, are all critical for the silencing of *ets-4* mRNA. This silencing correlates with the reduced levels of *ets-4* mRNA, consistent with its degradation. To test this further, we designed primers to indirectly monitor (by RT-qPCR) the *ets-4* mRNA cleavage (Figure 9A and B). We found that the levels of *ets-4* mRNA fragments spanning the REGE-1 cleavage site were as abundant in *rege-1(–)* mutants as in *rle-1(–)* or *rle-1(–); rege-1(–)* double mutants (Figure 9A). These results suggest that *ets-4* mRNA does not undergo REGE-1-mediated cleavage in the absence of RLE-1. We additionally performed the same analysis on the F1S reporters and found that the deletion of RCE had the same effect on the levels of *ets-4* mRNA fragments (spanning the REGE-1 cleavage site) as the deletion of ADE (Figure 9B). Taken together, our findings support a model where REGE-1 and RLE-1 (collectively referred to as R2) bind the *ets-4* 3′-UTR independently but collaborate to create a local microenvironment promoting, possibly with the help of additional factors, the *ets-4* mRNA cleavage by REGE-1 (Figure 9C).

## DISCUSSION

### The functional relationship between REGE-1 and RLE-1

We report here that REGE-1-mediated decay of *ets-4* mRNA also requires RLE-1/Roquin. Similar to Roquin, RLE-1 binds RNA via the interaction between its ROQ domain and the ADE-type SL. In contrast, the mechanism of RNA binding by REGE-1 remains an enigma. *In vitro*, REGE-1 and Regnase-1 display no RNA specificity [(9) and our observation]. *In vivo*, Regnase-1 was suggested to associate with RNA SLs, though this association could be indirect (6). A SL structure also appears to be important for REGE-1-mediated degradation, as REGE-1 cleaves the *ets-4* mRNA within the RCE SL. However, whether this or another SL is required for REGE-1’s binding remains to be determined.

Interestingly, Regnase-1 and Roquin-1 can interact directly and their interaction appears to be necessary for the regulation of some mRNAs (5). However, we have not been able to detect a similar association between REGE-1 and RLE-1. Moreover, a conserved (*sanroque*) residue of RLE-1 that in Roquin-1 is required for the association with Regnase-1 is dispensable for *ets-4* mRNA silencing. Finally, REGE-1 appears capable of binding *ets-4* in the absence of RLE-1. Together, these results suggest that RLE-1 facilitates REGE-1-mediated *ets-4* cleavage by a mechanism that does not rely on the recruitment of REGE-1. Among possible scenarios, RLE-1 binding could change the local conformation of *ets-4* RNA, facilitating REGE-1-mediated degradation by exposing the REGE-1 cleavage site. RBPs have been previously reported to stimulate local structural rearrangements of interacting mRNAs. One example is the association of Pumilio protein with the 3′-UTRs of target mRNAs. This association promotes a change in RNA structure that exposes either microRNA or RBP-binding sites, eventually leading to mRNA silencing (32,33). In another scenario, RLE-1 could affect REGE-1 protein to stimulate its RNase activity. Additional studies, which may require *in vitro* reconstitution of the RNA–protein complex, are needed to discriminate between these and other possibilities.

In mammals, Regnase-1 and Roquin can either cooperate in mRNA silencing or function independently of each other. While the former regulation was described in MEFs and T cells (4,5), the latter was reported in MEFs, macrophages and diverse cell lines (6,7). Thus, the choice between cooperative or independent regulation may depend on a particular cellular environment and/or mRNA target. At least some Regnase-1-independent functions of Roquin rely on the recruitment of the CCR4–NOT deadenylase complex (6,10). While this complex appears to be dispensable for the *ets-4* decay, RLE-1 could regulate additional mRNAs independently from REGE-1, particularly in tissues that express RLE-1 but not REGE-1. Pending the identification of additional RLE-1 targets, an interesting question for the future is whether their regulation may involve CCR4–NOT-mediated deadenylation.

### Evolution of REGE-1/Regnase-1-mediated mRNA silencing

In mammals, Regnase-1 degrades many mRNAs. In contrast, our analysis suggest that *ets-4* mRNA might be the sole target of REGE-1-mediated degradation. Such a high specificity could be explained by the need to precisely control the dosage of a master regulator controlling diverse ‘downstream’ effectors. In contrast to such hierarchical control, Regnase-1 (alone or with Roquin) appears to regulate the expression of multiple effectors in a more ‘horizontal’ fashion. For example, both REGE-1 and Regnase-1 regulate immunity, potentially reflecting a similar role for the ancestral protein. REGE-1 does so by controlling the expression of the master regulator ETS-4, which in turn induces transcription of various immunity genes (15). In contrast, Regnase-1 controls immunity by targeting mRNAs encoding diverse immune regulators, such as the cytokines interleukin-2 (IL-2), IL-6 and tumor necrosis factor (TNF), or T-cell co-stimulators ICOS and OX40 (3). It is possible that the exact functional relationship between REGE-1/Regnase-1 and RLE-1/Roquin-1 may reflect the type of gene network that they control: hierarchical in nematodes versus horizontal in mammals. In the former case, a concerted action of two factors recruited to mRNA independently of each other could result in a higher specificity and tighter regulation. Concluding, while REGE-1/Regnase-1 and RLE-1/Roquin-1 display a conserved functional relationship, the exact mechanisms underlying their cooperation appear to differ. Future studies of orthologous proteins in other species could shed more light on the evolution of this exciting mRNA silencing mechanism, potentially revealing a prototypic silencing mechanism involving both proteins.

## Supplementary Material

gkac609_Supplemental_File
