# Supplementary material for: The silencing of ets-4 mRNA relies on the functional cooperation between REGE-1/Regnase-1 and RLE-1/Roquin-1
Source: Nucleic Acids Res. 2022 Jul 12;50(14):8226–39. doi: 10.1093/nar/gkac609 (PMC9371910; doi:10.1093/nar/gkac609)
Supplement: gkac609_Supplemental_File [file gkac609_supplemental_file.pdf]

SUPPLEMENTAL INFORMATION

The silencing of *ets-4* mRNA relies on the functional cooperation between REGE-1/Regnase-1 and RLE-1/Roquin-1

Daria Sobańska[1], Alicja A Komur[1], Agnieszka Chabowska-Kita[1], Julita Gumna[1], Pooja Kumari[2], Katarzyna Pachulska-Wieczorek[1], and Rafal Ciosk [1, 2]

[1] Institute of Bioorganic Chemistry, Polish Academy of Sciences, Poznań 61-704, Poland

[2] Department of Biosciences, University of Oslo, Oslo 0316, Norway

SUPPLEMENTARY FIGURES

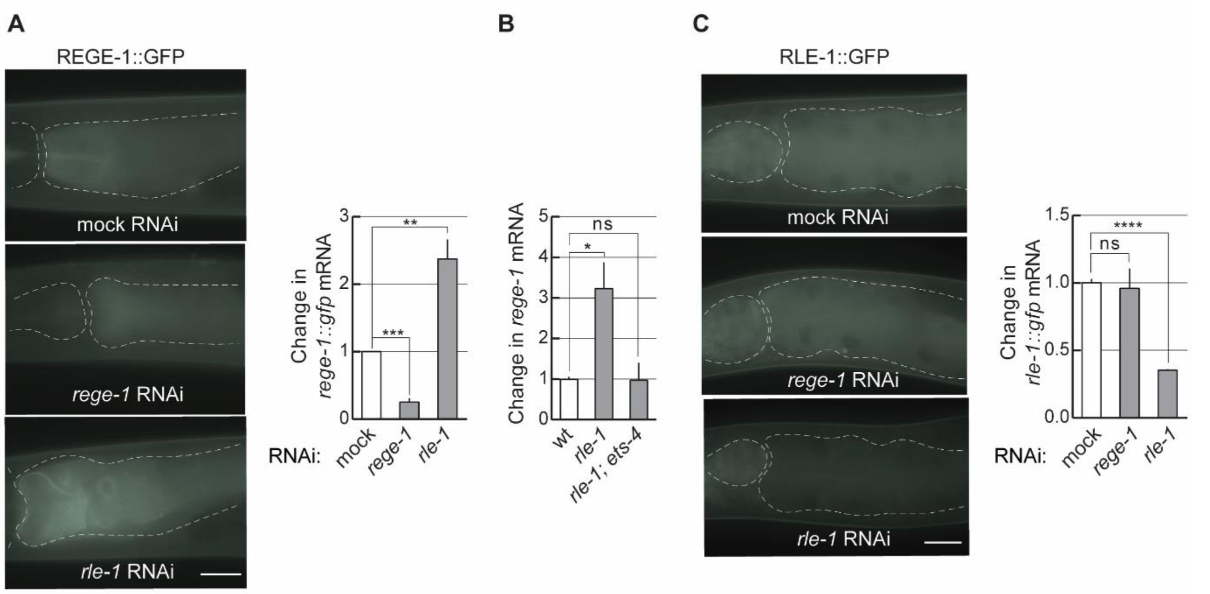

Figure S1. REGE-1 expression increases in the absence of RLE-1, while the expression of RLE-1 is not affected by the loss of REGE-1.

(A) Depletion of RLE-1 leads to REGE-1 upregulation. Left: partial view of representative live animals expressing rescuing REGE-1::GFP, subjected to either mock, *rege-1*, or *rle-1* RNAi, as indicated. To reduce gut-specific autofluorescence, the animals carried the *glo-1(zu391)* mutation (1). The pharynx and intestines are outlined. Scale bar: 20 µm. Right: The corresponding quantification of changes in *rege-1::gfp* mRNA measured by qRT-PCR. Bars represent the mean value from three independent biological replicates (N = 3). Error bars represent SEM; Two-tailed P-values were calculated using unpaired Student t-test using mock RNAi as a reference. ** indicates P ≤ 0.01, *** indicates P ≤ 0.001.

(B) REGE-1 upregulation in the absence of RLE-1 is caused by ETS-4. The level of *rege-1* mRNA was measured, by RT-qPCR, in animals of the indicated genotypes. Strains used: wt, *rle-1(rrr44)*, and *ets-4*(*rrr16*); *rle-1*(*rrr44*) double mutants. The mRNA levels were normalized to the levels of *tbb-2* (tubulin) mRNA. Two-tailed P-values were calculated using an unpaired Student t-test. Bars represent the mean value from four independent biological replicates (N = 4). Error bars represent SEM; * indicates P ≤ 0.05, “ns” = not significant.

(C) Depletion of REGE-1 does not impact the levels of RLE-1. The same as in A, except that animals expressed endogenous GFP-tagged RLE-1 and changes in *rle-1::gfp* mRNA were measured. Bars represent the mean value from three independent biological replicates (N = 3). Error bars represent SEM; Two-tailed P-values were calculated using unpaired Student t-test using mock RNAi as a reference. **** indicates P ≤ 0.0001.

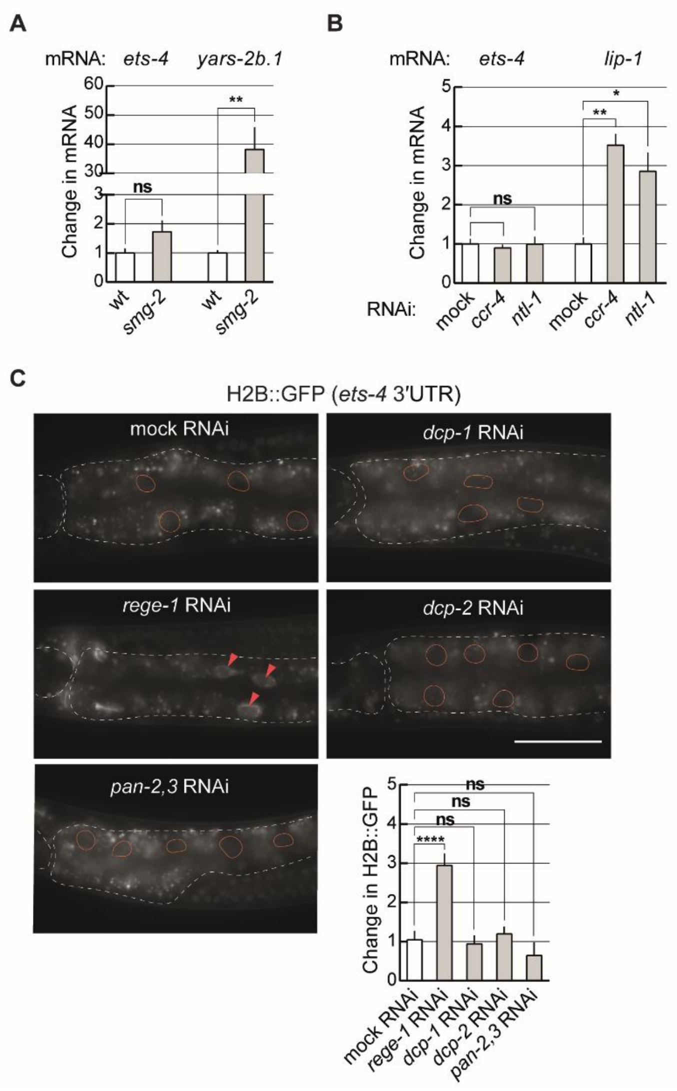

Figure S2. PAN-2 and PAN-3 deadenylases, as well as DCP-1 and DCP-2 decapping enzymes appear dispensable for *ets-4* silencing.

(A) The levels of *ets-4* and *yars-2b.1* (SMG-2 target (2)) mRNAs were measured, by RT-qPCR, in wt or *smg-2(rrr60)* animals. The mRNA levels were normalized to the levels of *tbb-2* (tubulin) mRNA. Two-tailed P-values were calculated using an unpaired Student t-test. Bars represent the mean value from three independent biological replicates (N = 3). Error bars represent SEM; ** indicates P ≤ 0.01, “ns” = not significant.

(B) The levels of *ets-4* and *lip-1* (CCR-4 and NTL-1 target (3)) mRNAs were measured, by RT-qPCR, in wt animals subjected to either mock, *ccr-4*, or *ntl-1* (components of the CCR4-NOT complex) RNAi, as indicated. The mRNA levels were normalized to the levels of *tbb-2* (tubulin) mRNA. Two-tailed P-values were calculated using an unpaired Student t-test. Bars represent the mean value from three independent biological replicates (N = 3). Error bars represent SEM; * indicates P ≤ 0.05, ** indicates P ≤ 0.01, “ns” = not significant.

(C) Depletion of additional *C. elegans* deadenylases and decapping enzymes has no impact on *ets-4* silencing. Upper left: Partial view of live animals, expressing the GFP::H2B reporter from a ubiquitous promoter (*dpy-30*), under the control of *ets-4* 3' UTR. Animals were subjected to either mock, *rege-1*

(positive control), *dcp-1, dcp-2* (decapping enzymes), or *pan-2,3* (other deadenylases) RNAi, as indicated. Ovals outline gut nuclei not expressing the reporter GFP, while arrowheads indicate gut nuclei in which the reporter is expressed. Scale bar: 50 μm. Lower right: Right: The corresponding quantification of changes in the reporter GFP intensity. Between five and ten nuclei per animal, in at least five animals per condition, were analyzed, to quantify GFP intensities from twenty-five nuclei in total (N = 25). Two-tailed P-values were calculated using an unpaired Student t-test. Bars represent the mean value from GFP intensity. Error bars represent SEM. **** indicates $P \leq 0.0001$; “ns” = not significant.

SUPPLEMENTARY TABLES

Table S1. The *C. elegans* strains used in this work.

**Genotype**	**CGC**/RAF
N2 Bristol	**N2**
smg-2(rrr60) I. [Q265].*	5097
rege-1(rrr13) I.	1657
rle-1(rrr44) III.	2055
rege-1(rrr13) I; rle-1(rrr44) III.	2102
rrrSi412 [Pdpy-30::gfp::h2b::ets-4 3'UTR; unc-119 (+)] II.;unc-119(ed3) III.	1740
rrrSi428[Pdpy-30::gfp::h2b::unc-54 3'UTR with transplanted (F1S) of ets-4 3`UTR; unc-119(+)] II; unc-119(ed3) III.	1844
rrrSi500[Pdpy-30::gfp::h2b::unc-54 3'UTR with transplanted mutated (F1SΔADE) of ets-4 3`UTR; unc-119(+)] II; unc-119(ed3) III.	5076
sybSi111*[Pdpy-30::gfp::h2b::unc-54 3'UTR with transplanted mutated (F1SΔRCE) of ets-4 3`UTR; unc-119(+)] II; unc-119(ed3) III.*	5113
glo-1(zu391) X.	**JJ1271**
glo-1(zu391) X.; rrrSi411 [Prege-1::rege-1(cDNA)::gfp::rege-1 3'UTR; unc-119(+)] II.	1761
glo-1(zu391) X.; rle-1(rrr58; syb1279) III.	5120
rrrSi431 [Prege-1::rege-1(cDNA [D231N, D313A, D314A, D332A])::gfp::rege-1 3'UTR; unc-119(+)] II.;unc-119(ed3) III.; rege-1(rrr13) I.	5046
rrrSi431 [Prege-1::rege-1(cDNA [D231N, D313A, D314A, D332A])::gfp::rege-1 3'UTR; unc-119(+)] II.;unc-119(ed3) III.; rege-1(rrr13) I.; rle-1 (rrr44) III.	5047
rle-1(rrr58) III; rege-1(rrr13) I.	5077
rle-1(rrr61; syb517) III.	5003
ets-4(rrr16) X.	1758
rle-1(rrr44) III; ets-4(rrr16) X.	5161
ets-4(rrr63; syb5507) X.	5173
rle-1(rrr59; syb5530) III.	5174
ets-4(rrr64; syb 5511) X.	5175
rle-1(rrr62; syb 5565) III.	5176
rege-1(rrr13) I; ets-4(rrr16) X.	1759

“CGC” indicates strain numbers deposited in the Caenorhabditis Genetics Center; “RAF” strain numbers in the Ciosk lab collection.

Table S2. DNA oligonucleotides used in this study.

Purpose	Name	Sequence
qPCR primers	qPCR_ets-4 F up	CTGAGAACCCGAATCATCCA
	qPCR_ets-4 R up	TCATTCATGTCTTGACTGCTCC
	qPCR_ets-4 F span	AAAGACAACGACGTGTTGCTATCTG
	qPCR_ets-4 R span	GACACAATAGGAATATGTTCTACAACG
	tbb-2 qPCR R	TGGTGAGGGATACAAGATGG
	tbb-2 qPCR F	GCTCATTCTCGGTTGTACCA
	GFP F	GTTGTCCCAATTCTTGTTGAATTAGATGG
	GFP R	TCGAGAAGCATTGAACACCA
	rege-1 qPCR F	CGGCAAATGAATGTTTATCCAG
	rege-1 qPCR R	ATCAGATCCAGTATTCACAGGTC
	B0252.1 qPCR F	TTGCATCAATGCGTCAACTACG
	B0252.1 qPCR R	CCTGCTCAGCTGTCTCTCG
	C01B10.6 qPCR F	CACTTCTCAAGCGACTACAGT
	C01B10.6 qPCR F	GGTCTAGTTGAACGGCTTGTTG
	nep-17 qPCR F	GCCTCCGTCAACCTATCAG
	nep-17 qPCR R	GGGAGAGGCGCAGTGCTG
	Y54G2A.11 qPCR F	GCAATGAGAAAAGCCCCGTT
	Y54G2A.11 qPCR R	TGGCGTCTGGATTTGGACTT
	131_lip-1_fw	TTCTCTCCCAGTTTCCGACC
	132_lip-1_rev	TCCGCCTTGATCTTCACCTT
	yars-2b.1-F	GTGGCCTTAAAAACGAGAAAG
	yars-2b.1-R	CAGCAGGTCCGTTGG
	qPCR_act-1_fw	CTATGTTCCAGCCATCCTTCTTGG
	qPCR_act-1_rv	TGATCTTGATCTTCATGGTTGATGG
unc-54 amplification for restriction enzyme cloning	unc-54 F XhoI	CTAGCTCGAGGTCCAATTACTCTTCAACATCC
	unc-54 R NotI	CTAGGCGGCCGCCAAAAAAATTTATCAGAAGTAAAAAAC
Il6 amplification for restriction enzyme cloning	Il6 403 F XhoI	CTAGCTCGAGTGCGTTATGCCTAAGCATATCAG
	Il6_NotI 1-403 R	CTAGGCGGCCGCTTTGTTTGAAGACAGTCTAAAC
ets-4 amplification for restriction enzyme cloning	ets-4 F XhoI	CTAGCTCGAGTCATCTGGCAGAAAGACAACGACGT
	ets-4 R NotI	CTAGGCGGCCGCAGATTATGAGACCTTTGGACTTG
	F1S F gibson	TTCTCTTAATTTCTTTGTGGTCAATACTCTGTTTACATTTTTC

F1S amplification for Gibson cloning	F1S R gibson	AAAGAAGCTAAAAAGGCGCGAGGAATATGTTCTACAACG
Ox40 amplification for restriction enzyme cloning	Ox40 F	CTAGCTCGAGGCATTACTAC
	Ox40 R	CTAGGCGGCCGCGCCAGTC
REGE-1 cDNA amplification for restriction enzyme cloning	REGE-1 F NotI	CTAGGCGGCCGCATGGATTCAACGGCTCGTGG
	REGE-1 R KpnI	GGTACCCTAGTCATTTTCGGTACTCTTTTTGAGC
Overlapping primers to create F1SΔRCE fragment	F1SdRCE F	TTCTCTTAATTTCTTTGTGGTCAATACTCTGTTTACATTTTTCAACTTGTCCTGTCGTTCAAGATATAC
	F1SdRCE R	AAAGAAGCTAAAAAGGCGCGAGGAATATGTTCTACAACGAACAGTACATGGAAGTATATCTTGAACGACAGGAC
Amplification of RLE-1 cDNA with tags	RLE-1 amp F pCS2	ATGGACTACAAAGACGATGACGACAAGATGGCGCCAACGGGTCAAGGTGGGC
	RLE-1 amp R pCS2	TCAcagatcctcttcagagatgagtttctgttcTCCCTCGACAGTCGGATTGAGATGAAGC
Amplification of REGE-1 cDNA with tags	REGE-1 amp F pCS2	ATGGACTACAAAGACGATGACGACAAGATGGATTCAACGGCTCGTGGCCAC
	REGE-1 amp R pCS2	TCAcagatcctcttcagagatgagtttctgttcTTTTCGGTACTCTTTTTGAGCTCGGATAATG
Amplification of N-FLAG, C-MYC sequence for Gibson cloning	FLAG gibs pCS2 F	CTACTTGTTCTTTTTGCAGGATCCCATATGGACTACAAAGACGATGACGACAAG
	MYC gibs pCS2	CTACGTAATACGACTCACTATAGTTTCAcagatcctcttcagagatgagtttctgttc
Amplification of templates for *in vitro* transcription	F1S F T7	TAATACGACTCACTATAGGGTCAATACTCTGTTTACATTT
	F1S R	AGGAATATGTTCTACAACGAACAG
	F1SdADE R	AGGAATATGTTCTACAAGGAATAG
	F1SdREGE R	AGGAATATGTTCTACAACGAACAG
SHAPE	ets-4 SHAPE	AGGAATATGTTCTACAACGAACAGT

All oligonucleotides used in this study were ordered from Merck, Germany.

SUPLEMMENTARY REFERENCES

1. Hermann, G.J., Schroeder, L.K., Hieb, C.A., Kershner, A.M., Rabbitts, B.M., Fonarev, P., Grant, B.D. and Priess, J.R. (2005) Genetic analysis of lysosomal trafficking in Caenorhabditis elegans. *Mol Biol Cell*, **16**, 3273-3288.
2. Son, H.G., Seo, M., Ham, S., Hwang, W., Lee, D., An, S.W., Artan, M., Seo, K., Kaletsky, R., Arey, R.N. *et al.* (2017) RNA surveillance via nonsense-mediated mRNA decay is crucial for longevity in daf-2/insulin/IGF-1 mutant C. elegans. *Nat Commun*, **8**, 14749.
3. Nousch, M., Techritz, N., Hampel, D., Millonigg, S. and Eckmann, C.R. (2013) The Ccr4-Not deadenylase complex constitutes the main poly(A) removal activity in C. elegans. *J Cell Sci*, **126**, 4274-4285.
